# Porcine transcriptome analysis based on 97 non-normalized cDNA libraries and assembly of 1,021,891 expressed sequence tags

**DOI:** 10.1186/gb-2007-8-4-r45

**Published:** 2007-04-02

**Authors:** Jan Gorodkin, Susanna Cirera, Jakob Hedegaard, Michael J Gilchrist, Frank Panitz, Claus Jørgensen, Karsten Scheibye-Knudsen, Troels Arvin, Steen Lumholdt, Milena Sawera, Trine Green, Bente J Nielsen, Jakob H Havgaard, Carina Rosenkilde, Jun Wang, Heng Li, Ruiqiang Li, Bin Liu, Songnian Hu, Wei Dong, Wei Li, Jun Yu, Jian Wang, Hans-Henrik Stærfeldt, Rasmus Wernersson, Lone B Madsen, Bo Thomsen, Henrik Hornshøj, Zhan Bujie, Xuegang Wang, Xuefei Wang, Lars Bolund, Søren Brunak, Huanming Yang, Christian Bendixen, Merete Fredholm

**Affiliations:** 1 Division of Genetics and Bioinformatics, IBHV, Grønnegärdsvej 3, The Royal Veterinary and Agricultural University, DK-1870 Frederiksberg C, Denmark; 2 Department of Genetics and Biotechnology, Danish Institute of Agricultural Sciences, Blichers Alle, DK-8830 Tjele, Denmark; 3 The Wellcome Trust/Cancer Research UK Gurdon Institute, Cambridge, CB2 1QN, UK; 4 Beijing Genomics Institute, The Airport Industrial Road, Beijing 101300, PR China; 5 Institute of Human Genetics, University of Aarhus, Nordre Ringgade 1, DK-8000 Aarhus C, Denmark; 6 Department of Biochemistry and Molecular Biology, University of Southern Denmark, Campus Vej 55, DK-5230 Odense M, Denmark; 7 Center for Biological Sequence Analysis, BioCentrum-DTU, Building 208, DK-2800 Lyngby, Denmark

## Abstract

**Background:**

Knowledge of the structure of gene expression is essential for mammalian transcriptomics research. We analyzed a collection of more than one million porcine expressed sequence tags (ESTs), of which two-thirds were generated in the Sino-Danish Pig Genome Project and one-third are from public databases. The Sino-Danish ESTs were generated from one normalized and 97 non-normalized cDNA libraries representing 35 different tissues and three developmental stages.

**Results:**

Using the Distiller package, the ESTs were assembled to roughly 48,000 contigs and 73,000 singletons, of which approximately 25% have a high confidence match to UniProt. Approximately 6,000 new porcine gene clusters were identified. Expression analysis based on the non-normalized libraries resulted in the following findings. The distribution of cluster sizes is scaling invariant. Brain and testes are among the tissues with the greatest number of different expressed genes, whereas tissues with more specialized function, such as developing liver, have fewer expressed genes. There are at least 65 high confidence housekeeping gene candidates and 876 cDNA library-specific gene candidates. We identified differential expression of genes between different tissues, in particular brain/spinal cord, and found patterns of correlation between genes that share expression in pairs of libraries. Finally, there was remarkable agreement in expression between specialized tissues according to Gene Ontology categories.

**Conclusion:**

This EST collection, the largest to date in pig, represents an essential resource for annotation, comparative genomics, assembly of the pig genome sequence, and further porcine transcription studies.

## Abstract 2

A resource consisting of one million porcine ESTs is described, providing an essential resource for annotation, comparative genomics, assembly of the pig genome sequence, and further porcine transcription studies.

## Background

The porcine genome has been characterized intensively through development of linkage maps, comparative maps, and physical maps [1,2] and Humphray and co-workers (unpublished data). These studies highlight the importance of genome research in pigs. Study of the porcine genome is important from the perspective of achieving sustainable breeding; also, the porcine model is an important research platform because of the anatomic, physiologic, biochemical, and metabolic similarities to humans. We recently showed that the evolutionary distance between the porcine and human genome sequences is smaller than the distance between mouse and human [3]. This provides a rationale for use of porcine sequences in gene expression comparisons with human and in transcriptome analysis of multiple tissues and organs [4,5] because, in contrast to human, there is easy access to tissues from the pig, including tissues from various embryonic developmental stages.

Here, we present an expression study based on 35 tissues represented by 98 cDNA libraries, of which 97 are non-normalized. For the assembly, more than one million expressed sequence tags (ESTs) were used, of which approximately two-thirds were generated in this study and the remaining one-third of ESTs are from public databases. The assembly not only contributes to identification of potential novel genes associated with specific tissues but it also allows us to address the key issue of gene expression structure in tissues. Furthermore, it is possible to search for genes that are expressed in a wide range of tissues, including genes that are of importance to embryonic development, because 24 of the 98 libraries used in the study are from various developmental stages.

Gene discovery and gene expression are key objectives of most genome projects, and consequently large-scale EST sequencing projects have been conducted for many organisms, including human, mouse, rat, chicken, frog, zebrafish, fruit-fly, and plants [6-22]. ESTs and full-length cDNAs provide direct information on the transcriptome and indirect information on the relation between the genome and different phenotypes. Because only about 25% of all protein-encoding mammalian genes have been characterized [23], a major current task in genomics is to characterize the functional importance of individual genes within the context of their interactions with other genes.

The transcriptome of a particular species can be analyzed by sampling a large number of ESTs from cDNA libraries, which are constructed from different tissues, or tissues from different developmental conditions or physiologic stages. Compared with characterization of normalized or subtracted cDNA libraries depleted of the most abundant transcripts, which optimizes discovery of novel genes [24-26], studies of non-normalized cDNA libraries are much more redundant but they provide raw information on the structure of gene expression levels [27].

To our knowledge the data presented here represent one of the largest collections of tissues ever included in a single EST expression study, and this makes it possible to conduct tissue-wise comparisons of the levels of expressed genes. Therefore, the generated pig EST resource represents an essential tool for the annotation and assembly of the forthcoming pig genome sequence, and it is a valuable resource for mammalian functional genomics research. The data presented here are also expected to have significant impact on efforts such as the Pig EST Data Explorer (PEDE) [28], which compiles full-length porcine cDNA sequences based on EST assembly. The resource makes it possible to compare coexpression patterns between organisms, for example between human, mouse, and pig. The PigEST resource, which is available online [29], contains a backend SQL database of clusters and singletons, as well as supplementary statical data files.

Below, we first describe the structure of gene expression in individual porcine tissues, and we find that the expression and the cluster sizes are scaling invariant. Then we show that brain and testes have greater gene diversity than any of the other tissues studied. Finally, we demonstrate that the established expression profiles represent the biologic function of the individual tissues.

## Results

### EST sequences and cDNA libraries

The analyses presented here are based on 1,021,891 porcine EST sequences, of which 636,516 were extracted from the Sino-Danish (SD) resource and 385,375 from GenBank [30]. These sequences were the result of initial rounds of cleaning (see Materials and methods, below). The SD EST sequences were generated from 98 cDNA libraries covering 35 tissues listed in Table 1 (97 non-normalized and 1 normalized [Pla]). For details concerning RNA extraction and library construction, see Materials and methods (below). No effort has been made to ensure that precisely matched cell populations are sampled from individual tissue when these tissues are represented by more than one cDNA library. Thus, two libraries representing the same tissue might, to some extent, differ in terms of expression.

### Accessing the porcine transcriptome: the assembly

The sequences were assembled using the 'Distiller package' [15] (see Materials and methods, below), resulting in 48,629 clusters and 73,171 singletons (single reads). The sequences from the SD resource are present in 35,344 contigs, of which 6,388 contigs were constructed solely from our resource. There are 13,285 contigs composed of public sequences that do not contain any of the ESTs generated in our resource, and in addition the public EST sequences are also present in 42,241 contigs. Hence, although there are about 380,000 public ESTs and 685,000 ESTs from the SD resource, the public sequences represent more genes than does our collection. This is to be expected because our data were generated from non-normalized libraries to provide raw expression profiles from different tissues, whereas many of the publicly available ESTs were generated from normalized libraries (for instance, see the reports by Hillier [31] and Scheetz [32] and their coworkers). This is also illustrated by the number of singletons. The SD resource yielded 26,429 singletons, whereas the public ESTs comprise 46,742 singletons. The Distiller assembly program also predicted 6,896 clusters to contain at least one chimeric sequence; such information can be useful when one is manually inspecting clusters. Furthermore, Distiller marked 430 clusters as groups of sequences linked by unknown or undetected repetitive elements, and about 2,500 clusters as representing alternative splice variants. These clusters were retained in the analysis conducted here. Single nucleotide polymorphisms (SNPs) are in part used by Distiller to phylogenetically decompose clusters into smaller clusters either of recently duplicated genes or, in some cases, of sequences originating from Chinese breeds and Danish breeds. In-depth analysis of SNPs combined with manual curation will be reported in another paper [33].

All expression patterns extracted in this work are based on comparing cluster sizes relative to library sizes (see Materials and methods, below), in conjunction with the extent to which a library is represented in a cluster. Fundamental to this process is the underlying distribution of expression, which we address as follows. We have sampled a large but non-exhaustive number of ESTs from each library (Table 1). Hence, we have sampled ESTs from each (non-normalized) cDNA library in the regime, where the absolute read count for each gene is proportional to the sampling size. Assuming that the assembly method can merge all reads from the same genes into the same cluster, then the cluster size is proportional to the total sampling size. This means that the cluster size within a single library is a measure of the expression of the corresponding gene it represents.

**Figure 1:**
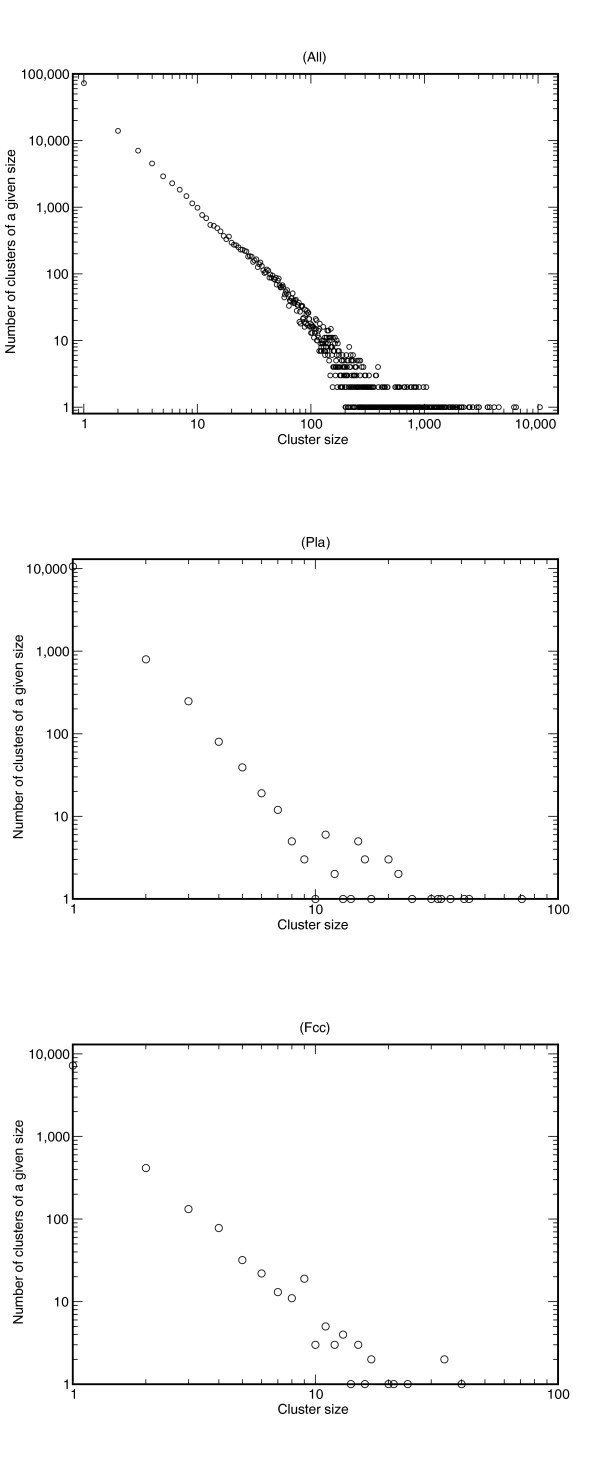
Distribution of cluster sizes. The number of clusters on the y-axis versus the cluster size (number of reads) on the x-axis exhibit a power law-like region. The distribution marked 'All' indicates the cluster size distribution for the entire dataset, whereas the other distributions are examples from specific libraries: 'Pla' (placenta, normalized) and 'Fcc' (cerebellum F100 days).

We considered the distribution of cluster sizes within each library (data not shown) and consistently observe, within one order of magnitude, that the number of cluster sizes is scaling invariant. Interestingly, this is also the case for the normalized library (Pla). When a library is normalized only the amount of highly expressed genes is reduced; the scaling properties are maintained (Figure 1). The normalized library has a steeper slope (on a log-log plot) than do the other libraries.

In agreement with the observation for each library, we also observe a scaling invariant cluster size distribution for clusters representing ESTs from libraries and public sequences, as shown in Figure 1. The slope is less than is observed for the individual libraries, which we interpret as resulting from the impact of the less redundant public sequences. As observed, even ESTs from normalized libraries exhibit scaling invariance, and when merging EST data from multiple sources one should still expect a scaling invariant distribution of cluster sizes, although they are no longer a direct measure of expression.

In essence, the scaling invariance tells us that large clusters are rare and small clusters are common, and that the few clusters that are already large tend to become larger when new reads are added to the pool of sequences being assembled. In a separate study we constructed a simple model of simulation of the assembly process that can lead to scaling invariance, and we also observe scaling invariance for EST assemblies from other organisms using other assembly methods, for example tgicl [34] (Schiebye-Knudsen and coworkers, unpublished data). Hence, only when one considers data from a non-normalized library does the cluster size (number of ESTs) for a gene correspond to the level of expression of that gene, and the slope is an approximate measure of the gene diversity of the library.

### Similarity match to existing sequences

To obtain information about contigs and single reads (hereafter referred to as 'conreads') from sequence similarity, the conreads were BLASTed against SwissProt and TrEmbl (UniProt 47.3) [35] for the protein search and against RNAdb (version August 2004), Fantom3, Rfam (version 7.0), and the MicroRNA registry (version 7.0) [36-39] for the noncoding RNA (ncRNA) search.

For protein comparison we searched for matches with high subject sequence (UniProt) coverage, as illustrated in Table 2. Here, we introduce various match levels from M0 to M5, where M0 is full-length subject coverage with at least 98% sequence identity. In Table 2, the number of sequences above the various match levels is indicated. From among the top 30 reported BLAST hits, we included the first match with the highest obtainable match level (which in the great majority of cases was the first reported BLAST match). For comparison with UniProt, we found 2,155 conreads with full subject coverage. We found 12,886 high confidence matches (M0 to M4) for contigs and 2,614 high confidence matches for the singletons.

When identifying ncRNAs, it is important to note that the RNA was enriched for poly(A) mRNA molecules by oligo(dT) selection. Interestingly, even though we masked for large and small ribosomal RNA and removed reads shorter than 100 nucleotides, we still observe small-sized RNAs such as tRNAs, U RNAs, and small structural elements. For example, some tRNAs are found in their surrounding sequence context. We also found 5.8S ribosomal RNAs as well as telomerase RNA. To obtain an indication of the amount of ncRNAs and elements of RNA structure (eleRNAs), an initial filtering was conducted up to level M2, counting each contig or singleton only once and ignoring tRNAs. The distribution for the match levels can be found in Table 2. The candidates at the match levels M0 and M1 were further curated, and we found 53 unique (non-tRNA) M0 or M1 matches to ncRNA or eleRNA, using in this case a minimum alignment length of 30 nucleotides. Hence, even good matches to mature microRNAs were ignored. The resulting matches are listed in Additional data file 1 (Table S1). Among the findings are 11 microRNA hairpins, of which four have already been predicted to be porcine microRNAs [3], and evidence of expression is hereby provided. None of these ncRNAs appears to have a particular tissue specificity.

### Gene diversity of the cDNA libraries

**Figure 2:**
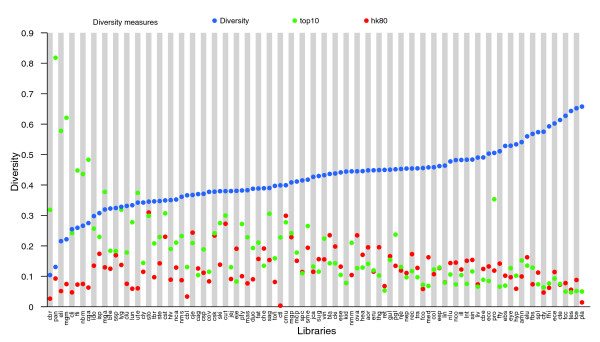
Diversity of cDNA libraries. The libraries (x-axis) are ranked according to their diversity (blue dot on y-axis). The names of the libraries on the x-axis correspond to those listed in Table 1. The diversity of a library is computed as the number of conreads in which the library has at least one read included, divided by the total number of reads present in the library. (See Materials and methods, in the text, for further details.) Two additional measures are included as well. 'top10' (green dots) refers to the fraction of reads comprising the 10 most expressed contigs in that particular library. 'hk80' (red dots) refers to the fraction of reads representing the 65 housekeeping candidates expressed in more than 80 libraries listed in Additional data file 1 (Table S2). Brain and testes libraries are among the most diverse. These also appear as the most diverse from the average diversity for each of the 35 tissues (not shown). Note that the normalized library Pla is among the most diverse tissues, as one would expect a normalized library to be.

The expression profiles of the individual SD cDNA libraries were examined to identify libraries containing the greatest number of different genes. That is, for each library we compared the number of conreads with the total number of reads from the library (after cleaning). We refer to ratio between these numbers as the 'diversity' of the library. Hence, high diversity means that only a small number of reads could be merged into contigs (low redundancy). In Figure 2 we show the rank of diversity. Brain and testes tissues are among the libraries with the greatest diversity, together with placenta, which was expected because it is a normalized library. The same observation is maintained when we average across libraries representing a tissue (data not shown) and is in agreement with reported observations in human and mouse [40,41] (and references therein). Among the libraries with low diversity, we find early developmental stages of liver and lung tissue as well as mammary gland, all three representing tissues with specialized and restricted functions. However, adult liver (Liv) has relative high diversity, reflecting a physiologically active tissue. These diversity observations appear to be in agreement with the observation of different slopes on the scaling invariant cluster size distributions for the libraries.

To further address the variance of the gene expression level in the different cDNA libraries, we also considered the percentage of reads being among the 10 most expressed contigs in a library. This is indicated by 'top10' in Figure 2. Although this measure indicates the variance of gene expression within a library, it is also relevant to compare the fraction of reads that are among the most common contigs, that is, those contigs that are expressed in a large number of libraries (housekeeping genes; see below). In Figure 2 'hk80' for each library indicates the fraction of reads that are part of a contig that is expressed in more than 80 libraries (in total 65 contigs; for details, see below). This measure makes it possible to compare expression levels of the genes relative to a common reference.

Not surprisingly, we observe that top10 to some extent correlates with the diversity, because a high top10 value correlates with low diversity. Furthermore, we also observe a slight decrease in hk80 for the libraries where the diversity increases. This is not surprising because increased diversity will cause the fraction of reads of any contig to decrease. However, the placenta (Pla) library has very low hk80, in agreement with the fact that it is a normalized library. There are other libraries with very low hk80, including Ctl, Cbr, and Cbl, all libraries that were discarded from the expression analysis because of bias in the data (see below). For the part of the libraries with low diversity, top10 is in general higher than hk80, which is in contrast to libraries with high diversity.

To ensure that the diversity was not an artefact resulting from the different library sizes, we compared the diversity against the library size and found that these do not correlate, as the Pearson's correlation coefficient has a value of -0.21. The impact of differences in diversity is further reflected in the number of BLAST matches for a given cDNA library. We investigated diversity as a function of the percentage of conreads that have a BLAST match (M0 to M4) and obtained a correlation coefficient of -0.64; this indicates that the greater the number of different genes expressed in a library, the larger the portion of them that appears to be novel or alternatively spliced (data not shown).

Differences in diversity are also reflected in the amount of common contigs between libraries. For example, a relatively large fraction of contigs from brain tissues can also be found in almost all other tissues, whereas only a small fraction of contigs from the other tissues is present in brain tissues (data not shown). In a number of cases we observed that more diverse libraries share a relative large portion of their expressed genes with all of the other libraries, whereas for less diverse libraries only a small portion of expressed genes are represented in the libraries with high diversity.

To ensure that the libraries had representative unbiased expression, we pruned a few libraries for which the fraction of (UniProt) matched contigs was unusually low. From the distribution of these fractions (Additional data file 1 [Figure S1]), the libraries Ctl, Cbr, Pan, Cbl, and Mgm are clearly separated in the low end from the remaining libraries, suggesting some problems with library construction (for instance, RNA degradation). This was further supported by manual inspection of the most highly expressed clusters for some of the libraries. For example, in the Ctl library the contig Ss1.1-rpigcf0_016260.5, which did not have any significant match to UniProt, appears to have a large number of reads clustered in the 5' end.

### Clustering of cDNA libraries and library gene content

The raw expression values (read count) were normalized with respect to library size and then with respect to the level of each contig across all libraries with accumulated values (see Materials and methods, below). For each pair of cDNA libraries we computed the euclidian distance between the expression values for all of the contigs represented with at least four reads in both libraries (see Materials and methods, below). A conservative cutoff of four reads ensured that significant expression was present in both libraries. This conservative cutoff yielded 4,776 contigs. Furthermore, for each pair of compared libraries, genes with expression values more than one standard deviation away from the center of mass value were also discarded. For each obtained value the average distance of all pairs was subtracted. These were clustered using the method of Eisen and coworkres [42] through the available software made by deHoon and coworkers [43]. We applied numerous combinations for hierarchical clustering. In general, we find that libraries from the following (adult) tissues cluster together: brain/spinal cord, testis, muscle/heart, and intestine. These tissues are represented by relatively many different genes either through high diversity of few libraries (brain/spinal cord and testis) or low diversity in many libraries (muscle/heart and intestine).

**Figure 3:**
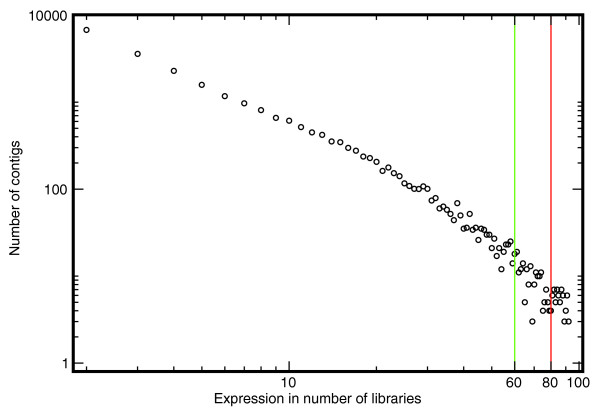
Distribution of cluster coverage of cDNA libraries. The values on the x-axis indicate the number of libraries for which there is at least one expressed sequence tag (EST) read present. The corresponding value on the y-axis shows the number of conreads for a given number of libraries. The vertical lines at 60 and 80 indicate cut-offs for potential housekeeping genes. The data indicate the presence of power law-like behavior. The data also show that we can only expect a small portion of the clusters to be composed of reads from many libraries.

To further analyze potential differential expression in normal tissues, we explored expression within the following three groups: brain/spinal cord, muscle/heart, and intestine. The main reason for considering only a fraction of the libraries and a fraction of the genes (expression subtables) is that relatively few gene clusters are represented in all libraries, as shown in Figure 3. We conducted a hierarchical clustering on data under the restricted requirement that all clusters must be represented in at least 10 libraries with at least four read counts (data not shown). From this it was possible to extract examples of genes with correlated expression such as the ribosomal proteins shown in Additional data file 1 (Figure S2 [A]). Furthermore, we found pairs of libraries sharing genes, in which the relative expression of the genes in the libraries is constant (see Additional data file 1 [Figure S2(B)] for an example).

**Figure 4:**
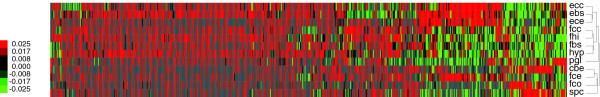
Patterns of differential expression. Differential expression within brain and spinal cord tissues. The clusterings were made using the package of de Hoon and coworkers [43], with options 'uncentered correlation' and 'average-linkage'. Gray fields indicate that the number of reads did not exceed the read cutoff of four reads for a given contig in a given library. However, such numbers were still counted as having the value zero when centering the expression values for the gene cluster. The tree has arbitrary scale.

Differential expression for the brain/spinal cord, muscle/heart, and intestine groups was also investigated using a more relaxed criterion of requiring a count of at least two reads, but requiring that gene clusters are present in at least 35% of the libraries. We also included expression from libraries constructed from tissues sampled at different developmental stages. This constituted the 658 genes in the brain/spinal cord group, 605 genes in the muscle/heart group, and 588 genes in intestine group, covering 1,231 different gene clusters (contigs). Within each of these groups expression for each gene cluster was normalized across all libraries (including absence of expression for a given library, counting it as zero) and hierarchical clustering was conducted for genes versus libraries. In all cases we find groups of genes that are differentially expressed between the different libraries. The brain/spinal cord group is shown in Figure 4. Note that the different libraries within the same or related tissues potentially represent different cell populations of these tissues and different physiologic stages. We see cases in which gene clusters are present in only a minor proportion of the libraries, and we observe clear cases of differential expression for each of these genes. Hence, we observe genes that are differentially expressed in normal tissues and between different developmental stages, for example cerebellum (Cbe and Fce). Similar types of observations were made for muscle/heart and intestine groups (data not shown).

The pattern of clustering in Figure 4 has a clear biologic explanation, which applies primarily when one is studying tissues such as brain, for which the sampling procedure from the different parts of the brain in the different developmental stages was well defined. In contrast, the sample collection from the intestinal tissues and muscles was not specifically defined; thus, in regard to some of the libraries the cellular components potentially vary considerably. In brain we see a clear pattern of clustering by developmental stage; for instance, Ecc, Ebs and Ece are clustered, and Fcc, Fhi and Fbs are clustered. These two groups of libraries correspond to different parts of the brain at developmental stage 50 days and developmental stage 107 days, respectively. The clustering was further inspected with respect to expressed genes. For instance CROC-4, a transcriptional activator of c-fos, was identified in the cluster of genes specific to the 50-day-old fetal brain libraries. This gene has been described as being expressed in early development of the brain and is involved in cell proliferation and differentiation [44]. Large numbers of ribosomal proteins and hypothetical proteins were also observed in these brain libraries. The high number of hypothetical proteins (novel genes) is in agreement with the observation of high gene diversity in the brain libraries. Also, in accordance with these observations, in general we observed a large amount of ribosomal proteins in the libraries from tissues sampled in early developmental stages (data not shown).

As mentioned above, not all gene clusters are represented in all libraries. This complicates expression analysis, but it is of interest when describing how many libraries are represented in a given gene cluster (given that the EST sequences were sampled from non-normalized libraries). As indicated in Figure 3, we observe scaling invariant-like behavior, which is in agreement with the scaling invariance observed for cluster sizes (Figure 1). In essence, clusters with reads from a large number of libraries are much more rare (by order of magnitude) than are clusters with reads from a small number of libraries. Therefore, considering a cluster by chance it is more likely to find it expressed in only a few libraries. The really large clusters that deviate from this behavior could be due to extraordinarily high levels of transcription (represented by housekeeping genes) and alternative splice variants being merged into the same cluster. For example, we find only 65 contigs to be expressed in more than 80 libraries (of the 92 remaining after cleaning), as shown in Additional data file 1 (Table S2). Approximately 40% of these genes are ribosomal proteins, which is not surprising because they are essential components of the cellular machinery. Clearly, these genes can be considered housekeeping genes, but the scaling invariant-like behavior shown in Figure 1 makes it clear that genes expressed in many libraries are less likely to be sampled from all libraries (with the current EST sampling strategy). We therefore underestimate the number of housekeeping genes, when these housekeeping genes are defined as genes present in all libraries at some minimum level of transcription.

### Library-specific genes

We (conservatively) selected cDNA library-specific gene candidates as follows. The libraries with the two highest levels of expression for a given transcript were compared, and it was required that the library with the highest level of expression had a read count of at least 10 reads, regardless of the library size. Then, we computed the probability of observing the counts for the highest expression value, given the observed counts of the second highest using the work of Audic and Claverie [45], and required this to be less than 0.05 (see Materials and methods, below). In cases in which only a single library had high expression, the 'second highest' count was set to zero whereas the library size was set to the smallest library size in the dataset.

We obtained a list of 876 gene clusters, to which we assigned corresponding BLAST matches if available; 676 of these gene clusters had a match of at least M4 to UniProt. The top 50 in the list is shown in Additional data file 1 (Table S3); the complete list is available online via the download area of the PigEST resource [29]. Note that this list contains genes that can be specific in one of multiple libraries from the same tissue. This can be explained by the fact that no effort has been made to ensure sampling of precisely matching cell populations from the individual tissue. The expression profile of the genes belonging to the top 50 'tissue-specific gene list' listed in Table 3 was manually inspected and compared with UniGene at NCBI [46]. The gene names used in UniGene were those from the description line of the best matching BLAST hit (regardless of the match level) of the top 50 list (Additional data file 1 [Table S2]). The comparison was done with human data when available and otherwise with mouse or cattle data. In 25 cases the tissue (in which a given library specific gene was found) in which the corresponding gene was highly expressed was in agreement with the public data. In 14 cases there was not enough expression data to draw any conclusions. In 11 cases the published results did not agree with our findings. However, considering only the most confident BLAST matching contigs (M0 level only), 16 cases agreed with UniGene, four cases had insufficient data, and six did not agree. Hence, for more than 70% (16/22) of UniGene matching M0 cases, we found agreement in expression.

We also observed three cases in which the discrepancies pertain to expression of liver-specific genes in the Lun library, which points toward a possible contamination of this library with liver. Further inspection of the library confirms the presence of high levels of liver-specific genes. Furthermore, real time quantitative polymerase chain reaction (qPCR) analysis was performed on eight selected genes from additional in-house tissue (see Materials and methods, below). These genes were as follows: pepsinogen c, vitamin D-dependent calcium-binding protein, fetuin B, gastrotropin, pepsinogen A precursor, myelin basic protein, surfactant protein-C, and troponin C (also see Materials and methods, below). In seven cases the qPCR results were in agreement with *in silico *results. In one case (the gastrotropin gene [Ss1.1-rill310b_f20.5; Table 3]), the greatest expression was found in thymus, which in our *in silico *study was positioned as the second tissue in which this gene is most highly expressed.

### Expression characterization by top level Gene Ontology terms

To address the issue of functional representation of each library, we constructed Gene Ontology (GO) profiles [47] at the top level for each of the three main classes: 'molecular function', 'cellular component', and 'biological process'. The roughly 10,000 conreads that had a BLAST match to UniProt of M0 to M3 were analyzed. Profiles were constructed for each library and compared in terms of the fraction of expression content for each of the top categories within the three classes. Profiles for each cDNA library can be found in Additional data files 2 to 4, in which the normalized library Pla (Placenta) is included for comparison. On initial inspection the content of most libraries appears uniform. However, when the libraries are compared in greater detail differences are found. For each library we computed the log odds ratio (in bits) between the observed fraction of a subcategory (for instance, 'binding' within 'molecular function') and the average fraction taken over all libraries. Heat maps were constructed for each of the main categories.

**Figure 5:**
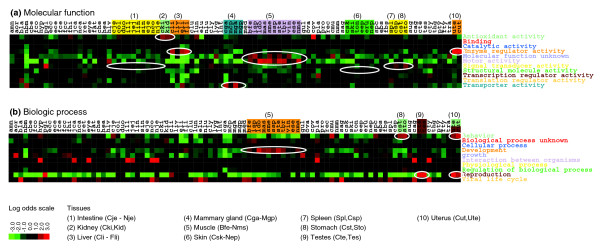
Gene Ontology content of cDNA libraries and tissues. A heat map of the log odds values (in bits) for each library, found by comparing the observed fraction of the Gene Ontology top level categories of **(a) **'molecular function' and **(b) **'biological process' with the respective averages. Gene Ontology categories were taken from corresponding M0 to M3 BLAST matches to UniProt. The libraries are grouped by their corresponding tissues, and the coloring indicates the category where we find higher expression than by chance. Only the relevant tissues are indicated by numbers and listed by their range of cDNA library names.

In Figure 5 heat maps for 'molecular function' and 'biological process' are shown. The libraries are ordered as in Table 1, and libraries within the same tissue are listed adjacent to each other. Figure 5a shows log odds ratios for 'molecular function'; we observe that some tissues have over-representation of some categories, as indicated by the coloring and ellipsoids. We find that muscle libraries have an over-representation of the category 'motor activity', and almost all other libraries are under-represented in this category. Muscle libraries are also clearly over-represented in the category 'molecular function unknown'. The liver and uterus libraries are clearly over-represented in the categories 'enzyme regulator activity'. Kidney is over-represented in the category 'antioxidant activity'. The mammary gland libraries are over-represented in the category 'transporter activity'. Finally, skin libraries are (slightly but consistently) over-represented in the 'structural molecule activity' category.

These findings are in agreement with the molecular function of the individual tissues, confirming that the expression profiles of the libraries are in agreement with the physiology and the function of the individual tissues. Furthermore, the two categories that constitute approximately 50% of expression (Additional data file 2), namely 'binding' and 'catalytic activity', do not in general vary much relative to their average fraction. It is worth noting that all of the tissues exhibiting over-representation of a specific GO category are tissues that in general do not have very high diversity and tissues that also have relatively specific functions.

For the other main GO category, 'biological process' (Figure 5b), we made the following observations. With regard to 'biological process', tissues such as testes and uterus are highly over-represented in the category 'reproduction', and almost all other libraries are highly under-represented. Muscle libraries are over-represented in the 'development' category, and stomach and uterus are over-represented in the 'behavior' category. For the main GO category, namely 'cellular component' (not shown), we find that fat and skin are over-represented in 'extracellular matrix'. A direct correlation between expression and function is clear in regard to 'reproduction' and 'extracellular matrix'. However, because some of the categories are quite broadly defined, a direct correlation to tissue physiology is not clear-cut for all categories.

Furthermore, we also looked for correlations between expression patterns, and we found a handful of cases of correlating categories (|cc| > 0.63); examples are shown in Additional data file 1 (Figure S3) for 'transcription regulator activity' and 'translation regulator activity'. Other examples include 'physiological process' and 'regulation of biological process' as well as 'binding' and 'structural molecule activity' (data not shown). Similar correlations are observed for other classes as well, for example the categories 'cellular process' and 'physiological processes' are strongly correlated in the 'biological process' class.

## Discussion

We present a resource of more than one million porcine EST sequences, of which two-thirds were generated for the work presented here and the remaining one-third of EST sequences were extracted from public databases. The sequences from our PigEST resource were extracted from 97 non-normalized cDNA libraries and one normalized cDNA library representing in total 35 tissues. We have conducted an initial expression analysis, providing novel insight into the structure of a large-scale set of EST sequences from non-normalized libraries of normal tissue and tissue sampled at various developmental stages. The assembly resulted in approximately 48,000 contigs and 73,000 singletons. Out of these a total of 2,155 contigs was identified with full-length coverage and high confidence match to UniProt; in addition, 12,886 contigs and 2,614 single reads were found with high confidence matches. Thus, using stringent criteria, about 25% of our contigs and singletons were matched with high confidence. In addition, we also identified approximately 50 noncoding RNAs, including 11 microRNAs (of which four were found previously in genomic sequence [3]).

We conducted an extensive analysis of gene expression structure in tissues. We found that the assembled clusters led to the observation that expression and the cluster sizes are scaling invariant, an observation that is in agreement with the observed scaling invariance of microarray expression data (for example see the reports by Hoyle [48] and Lu [49] and their coworkers). The results indicate that large clusters (high expression) are rare, whereas small clusters (low expression) are common.

To identify the tissues containing the most different genes, we compared library sizes with the number of conreads. We found that the tissues brain and testes have higher gene diversity than the other tissues. This is in accordance with the observation that the most diverse libraries have steep slopes in their scaling invariant cluster size distributions. Furthermore, in agreement with this, we observed that high-diversity tissues in general have a lower percentage match to UniProt, indicating that libraries representing these tissues are suitable for finding novel genes or alternatively spliced genes (not assembled into the same contig).

A major challenge was to extract meaningful expression patterns from the EST libraries. To provide the most reliable starting point for these studies, we removed libraries with biased expression patterns. It is most likely that there are still a few artificial expression patterns in the remaining clusters, in particular among the genes in the tissue-specific gene list. However, we found that only a constant fraction of gene clusters (contigs) can be expected to be present in all libraries. The fact that not all gene clusters are represented in all libraries complicates the differential expression analysis across the libraries. Nevertheless, we showed that meaningful expression results could be extracted from the resource. First, we showed that tissues represented by many libraries or libraries with high diversity in general cluster together. Second, we found differential expression patterns within the tissues brain/spinal cord, muscle/heart, and intestine. These tissues are represented by high-diversity or multiple libraries involving approximately 1,200 gene clusters. In these libraries, manual inspection revealed that the expression patterns of specific genes were in accordance with the biological function of the individual tissues.

Furthermore, we extracted 876 gene clusters as candidates for cDNA library specificity, of which approximately 7% were in libraries from a given developmental stage. The top 50 candidates were inspected manually, and it was found that our results agreed with most of the corresponding expression profiles available via UniGene (25 versus 11; 14 cases did not have similar data). However, these numbers were 16 versus 6 for the best BLAST matching contigs (M0 level). We also conducted qPCR in eight selected genes, and found that their expression profiles agreed with the corresponding expression found in the EST study.

We also considered how often the contigs were expressed in a given number of cDNA libraries. The distribution of these data reveals a scaling invariant-like behavior, which appears to be a novel observation. Thus, there is a fixed proportion between the number of genes (contigs) expressed in many libraries and those expressed in few. Hence, considering a small number of libraries and tissues, there is no guarantee that genes expressed in all libraries will also be expressed in all libraries of a bigger sample. However, because we used 92 libraries covering 32 tissues in this study (poor quality and normalized libraries being discarded), we cover a large proportion of all existing tissues. Therefore, we have high confidence in our housekeeping gene candidates list. It should, however, be noted that because of the scaling properties, we are probably missing many housekeeping genes, in particular those expressed only at low levels.

We have found that the expression in related tissues correlates strongly and provided examples of correlation of expression between pairs of libraries sharing the same genes, indicating functional relationships. When analyzing the portion of the data (about 10,000 contigs and single reads) with good match to existing proteins in UniProt, we extracted meaningful GO assignments of the libraries. For example, we found that muscle libraries are over-represented in the 'motor activity' category of 'molecular function', and that testes and uterus libraries are strongly over-represented in the 'reproduction' category of 'biological process'. Interestingly, the muscle libraries contain the highest relative amount of genes with poor annotation, implying that the relative proportion of functionally unaccountable genes is higher than in any other tissue.

There are many obvious directions in which this work can be continued. It is relevant, for instance, to conduct a comparison with porcine UniGene [46]. More detailed studies of expression profiles will provide new information about the mammalian transcriptome and will provide new functional information with regard to individual non-annotated transcripts. As expected, sequencing of non-normalized cDNA libraries has resulted in a high level of redundant transcripts. It has, however, also resulted in new information about diversity in individual tissues. Because the greatest diversity is found in brain and testes, it is clear that additional sequencing of ESTs from these tissues is expected to provide novel transcripts.

In conclusion, we have not only demonstrated that the established expression profiles not only represent the biologic function of the individual tissues, but also we have provided novel information about the gene expression structure of the tissues. This resource [29] will be of importance for comparative transcriptomics, annotation of novel genes, and systems biology.

## Materials and methods

### Construction of the 98 porcine cDNA libraries

#### Tissue collection

Tissues were collected from 200 pigs used in the Danish pig production industry; breeds were cross-breeds from Landrace, Yorkshire, Duroc, and Hampshire. A Chinese breed, Taihu/Erhualian, was also used. Tissues were immediately frozen in liquid nitrogen after sampling and were stored at -80°C until use. Some of brain tissues were kept in RNA buffer (Ambion, Cambridge, UK) in order to prevent degradation.

#### RNA extraction

Total RNA was extracted from up to 1 g of the various pig tissues using TRI REAGENT (Molecular Research Center, Inc.) or RNeasy (Qiagen, GmbH, Germany), following the manufacturers' protocols. Quality of the extracted total RNA was assessed by agarose gel electrophoresis. PolyA+ mRNA was isolated from 0.1 to 1 mg total RNA using polyATtract mRNA isolation system IV (Promega, Madison, USA) or Oligotex mRNA Purification System (Qiagen), and approximately 0.5 μg of polyA+ mRNA was quality checked by agarose gel electrophoresis

#### cDNA library construction

Directional cloneable cDNA was synthesized from 5 μg Poly(A+) mRNA using the cDNA Synthesis Kit (Stratagene, Cedar Creek, USA) following the manufacturer's protocol. The cDNA was size fractionated using Sepharose CL-2B, as included in the library kit, or by agarose gel electrophoresis followed by purification using Qiaex II Gel extraction kit (Qiagen). Purified cDNA was ligated into *Eco*RI/*Xho*I digested pBluescript II XR (Stratagene) or pTrueBlue (Alert B&C, Quebec, Canada) using temperature-cycle ligation [50] followed by PCR validation of the ligation reaction. The ligation product was precipitated and electro-transformated into *E. coli *XL1-Blue MRF' (Stratagene) and plated on blue/white selective LB agar, and positive clones were picked into 2xTY (100 μg/ml Amp, 10% [vol/vol] glycerol) in 384-well plates using a QPix2 robot (Genetics Limited, Norwich, UK), incubated for 24 hours at -37°C, and stored at -80°C until use. The insert length of each library was evaluated in 192 clones by PCR. Quality criteria was set at a maximum of 8% colonies without insert and a maximum of 10% to 20% with inserts less than 400 base pairs (with the exception of the brain libraries, for which the latter was set at 20%).

The RNA used in the libraries Cag, Cga, Che, Cje, Cki, Cli, Cly, Cmu, Cov, Csk, Csp, Cst, Cte, Cov, and Cut was extracted from Chinese pigs using TRI REAGENT (Molecular Research Center, Inc.). The RNA corresponding to these libraries were sent to Denmark and the libraries were made. The RNA was EtOH precipitated on arrival. RNA quality was checked by optic density (OD) and agarose gel. The RNA corresponding to the libraries Cli, Cga, Cov, Cly, Csp, and Cag contained genomic DNA, and it was re-extracted using TRI REAGENT before proceeding to the construction of the libraries in order to eliminate the gDNA. The library construction and quality criteria are as described before. In total 98, cDNA libraries covering 35 tissues were constructed (Table 1).

#### EST sequencing

T3 primer was used from the polylinker of the vectors to sequence the 5' end of each insert using standard protocols. Sequencing reactions were analyzed on the MegaBACE 1000 DNA Analysis System (Amersham Bioscience, Buckinghamshire, UK).

#### qPCR experiments

In order to compare *in silico *based expression with qPCR expression in tissues from a new set of pigs, we had to select genes within the top 100 list to match tissue combinations from the currently available in-house cDNA panel. Eight genes were selected: pepsinogen c, vitamin D-dependent calcium-binding protein, fetuin B, gastrotropin, pepsinogen A precursor, myelin basic protein, surfactant protein-C, and troponin C. See corresponding contig names in Table 3. The gene expression levels of these eight pig genes were measured using real-time qPCR. A cDNA panel of 18 different pig tissues (bone marrow, liver, thymus, kidney, stomach, jejunum, muscle, heart, cerebellum, cortex cerebri, hippocampus, lung, pancreas, skin, bladder, lymph, testis, and ovary) from three Landrace piglets was used for qPCR studies. The Ct values were normalized with a normalization factor generated from three reference gene's expression ratios (ribosomal protein L4 [RPL4]; hypoxanthine phosphoribosyltransferase 1 [HPRT1], and β-actin [ACTB]) and calculated using geNorm [51].

The Primer3 software [52] at MACROBUTTON HtmlResAnchor [53] was used for primer design. Two primers were designed covering around 100 base pairs of each cDNA for the eight selected genes and the three reference genes (Table 3). A standard curve was constructed using the purified PCR product generated for each specific primer pair. Single reactions were prepared for each cDNA, along with the standard curve and a nontemplate control using the Brilliant r SYBR r Green Master Mix (Stratagene). Each reaction consisted of 20 μl containing 2 μl of one-eighth diluted cDNA and 5 to 20 pmol of each primer. The real-time reverse transcription PCR was performed using a Mx3000 detection system (Stratagene). The cycling conditions were one cycle of denaturation and hot start at 95°C/15 min, followed by 40 cycles of amplification (95°C for 30 s, 60-63°C for 1 min, 72°C for 30 s) and one three-segment cycle of product melting (95°C for 1 min, 55°C for 30 s, and 95°C for 30 s). The baseline adjustment method of the Mx3000 (Stratagene) software was used to determine the crossing thresholds (Ct).

### Data extraction and cleaning

In total, 970,404 raw chromatogram files were generated in the SD resource. Those meeting the criteria were uploaded at the NCBI trace archive and are avaliable upon searching using the center name 'SDJVP' (Sino-Danish Joint Venture Project) and dates from 16 July to 31 July 2006. The contiguous ranges of accession numbers are summarized in Additional data file 1 (Table S4).

The plate archives and files were examined for duplicates, misplaced and erroneously packed plate archives, and erroneously named trace files. Where possible naming errors were corrected, and for different reads with identical names the latest generated were chosen over earlier ones. Here, the 970,404 raw chromatogram files were processed using phred (-trim alt "" -trim cutoff 0.01) and phd2fasta [54,55], which yielded 823,871 files containing sequences. Then vector sequences from Univec (Kitts and coworkers, unpublished data) were removed along with linker (*Xho*I) sequence. Resulting sequences with length less than 100 nucleotides were also removed. This resulted in 685,851 reads. These were repeatmasked in the assembly process using the Distiller package (see below).

The databases used for repeatmasking and vector cleaning were from Univec, namely ribosomal sequences 18S pig (gi 52694694) and 28S human (gi 337381). The latter because there were no full 28S sequence available for pig in GenBank [30]. The porcine mitochondrial genome sequence (gi 33320837) was included as well. Finally, in order to remove repetitive segments the following libraries from RepBase were used [56]: simple.lib, alu.lib, at.lib, carnivorecut. lib, carnivore.lib, cetartiocut.lib, cetartio.lib, cut1.lib, cut2.lib, humlines.lib, humsines.lib, humspec.lib, l1.lib, mir.lib, mirs.lib, othermamreps.lib, retrovirus.lib, rod1.lib, rod2.lib, rodcut2.lib, rodcut.lib, and rodcutsines.lib. Removing repeatmasked sequences from the dataset reduced the dataset to 636,516 sequences.

The dataset was extended with 398,837 EST sequences downloaded from GenBank (the Entrez nucleotide database) [30], searching Organism and EST Database Division using the following terms: 'Sus scrofa[ORGN]' AND 'gbdiv est[PROP]'. After cleaning, this set was reduced to 385,375 sequences. Hence, in total 1,021,891 EST sequences were analyzed.

### Assembly

To assemble the EST sequences we used the Distiller assembly program, which was used in another large-scale EST project conducted in *Xenopus tropicalis *[15] (see details in that report). Briefly, the Distiller program first conducts a pair-wise comparison of all sequences using BLAST. Then, sequences are clustered, but with a requirement of double linkage for sequences added to the clusters. This lowers the chance, for example, of mis-assembly of ESTs from two genes into a single cluster through a chimeric sequence. In a later stage, clusters are joined using a more relaxed linkage criteria. Consensus sequences are constructed from sets of adjacent 12-mers over the aligned sequences in the given region. After a first round of assembly, the clusters were phylogenetically decomposed to separated close gene family members. Furthermore, Distiller detects SNPs, alternative splice variants, and chimeric sequences. At an early stage in the project, from among other assembly programs, we also applied the tgicl package [34]. This was applied before inclusion of the public sequences, and the observations on diversity and pair-wise correlations of GO categories were essentially the same as presented in the Results section (above). However, because of the features of Distiller, including the double linkage clustering and alternative splicing predicting (applied elsewhere), we found it more suitable to apply this program here.

### Expression levels of genes

The relative expression of gene (cluster/singleton) *i *in library *j *is computed as *x*_*ij *_= *n*_*ij*_/(*N*_*j*_*M*_*i*_), where *n*_*ij *_is the number of read sequences in gene *i *from library *j*, Nj=∑i=1#genesnij
 MathType@MTEF@5@5@+=feaafiart1ev1aaatCvAUfeBSjuyZL2yd9gzLbvyNv2Caerbhv2BYDwAHbqedmvETj2BSbqee0evGueE0jxyaibaiKI8=vI8tuQ8FMI8Gi=hEeeu0xXdbba9frFj0=OqFfea0dXdd9vqai=hGuQ8kuc9pgc9s8qqaq=dirpe0xb9q8qiLsFr0=vr0=vr0dc8meaabaqaciGacaGaaeqabaqadeqadaaakeaacaWGobWaaSbaaSqaaiaadQgaaeqaaOGaeyypa0ZaaabCaeaacaWGUbWaaSbaaSqaaiaadMgacaWGQbaabeaaaeaacaWGPbGaeyypa0JaaGymaaqaaiaacocacaWGNbGaamyzaiaad6gacaWGLbGaam4CaaqdcqGHris5aaaa@435F@ is the number of reads in library *j *(after running phred and repeatmasker, and so on), and Mi=∑j=1#libsnij/Nj
 MathType@MTEF@5@5@+=feaafiart1ev1aaatCvAUfeBSjuyZL2yd9gzLbvyNv2Caerbhv2BYDwAHbqedmvETj2BSbqee0evGueE0jxyaibaiKI8=vI8tuQ8FMI8Gi=hEeeu0xXdbba9frFj0=OqFfea0dXdd9vqai=hGuQ8kuc9pgc9s8qqaq=dirpe0xb9q8qiLsFr0=vr0=vr0dc8meaabaqaciGacaGaaeqabaqadeqadaaakeaacaWGnbWaaSbaaSqaaiaadMgaaeqaaOGaeyypa0ZaaabCaeaacaWGUbWaaSbaaSqaaiaadMgacaWGQbaabeaakiaac+cacaWGobWaaSbaaSqaaiaadQgaaeqaaaqaaiaadQgacqGH9aqpcaaIXaaabaGaai4iaiaadYgacaWGPbGaamOyaiaadohaa0GaeyyeIuoaaaa@451C@ is the accumulated expression of gene *i*. The number of genes (#genes) is the number of conreads and the number of libraries (#libs) is usually 92, which is the number of libraries obtained after cleaning. In cases in which subsets of libraries are considered, *#libs *is the number of the libraries in such a subset.

The distance between two libraries (*j *and *k*) is computed as d=∑i(xij−xik)2
 MathType@MTEF@5@5@+=feaafiart1ev1aaatCvAUfeBSjuyZL2yd9gzLbvyNv2Caerbhv2BYDwAHbqedmvETj2BSbqee0evGueE0jxyaibaiKI8=vI8tuQ8FMI8Gi=hEeeu0xXdbba9frFj0=OqFfea0dXdd9vqai=hGuQ8kuc9pgc9s8qqaq=dirpe0xb9q8qiLsFr0=vr0=vr0dc8meaabaqaciGacaGaaeqabaqadeqadaaakeaacaWGKbGaeyypa0ZaaOaaaeaadaaeqbqaaiaacIcacaWG4bWaaSbaaSqaaiaadMgacaWGQbaabeaakiabgkHiTiaadIhadaWgaaWcbaGaamyAaiaadUgaaeqaaOGaaiykamaaCaaaleqabaGaaGOmaaaaaeaacaWGPbaabeqdcqGHris5aaWcbeaaaaa@418D@, summing only over genes expressed (above some threshold; typically three reads) in both libraries. Similarly, when computing Pearson s correlation coeffcient r=rik/riirkk,rjk=∑i(xij−μj)(xik−μk), with μj=(∑ixij)/#genes
 MathType@MTEF@5@5@+=feaafiart1ev1aaatCvAUfeBSjuyZL2yd9gzLbvyNv2Caerbhv2BYDwAHbqedmvETj2BSbqee0evGueE0jxyaibaiKI8=vI8tuQ8FMI8Gi=hEeeu0xXdbba9frFj0=OqFfea0dXdd9vqai=hGuQ8kuc9pgc9s8qqaq=dirpe0xb9q8qiLsFr0=vr0=vr0dc8meaabaqaciGacaGaaeqabaqadeqadaaakeaacaWGYbGaeyypa0JaamOCamaaBaaaleaacaWGPbGaam4AaaqabaGccaGGVaWaaOaaaeaacaWGYbWaaSbaaSqaaiaadMgacaWGPbaabeaakiaadkhadaWgaaWcbaGaam4AaiaadUgaaeqaaaqabaGccaGGSaGaamOCamaaBaaaleaacaWGQbGaam4AaaqabaGccqGH9aqpdaaeqaqaaiaacIcacaWG4bWaaSbaaSqaaiaadMgacaWGQbaabeaakiabgkHiTiabeY7aTnaaBaaaleaacaWGQbaabeaakiaacMcacaGGOaGaamiEamaaBaaaleaacaWGPbGaam4AaaqabaGccqGHsislcqaH8oqBdaWgaaWcbaGaam4AaaqabaGccaGGPaaaleaacaWGPbaabeqdcqGHris5aOGaaiilaiaabccacaqG3bGaaeyAaiaabshacaqGObGaaeiiaiabeY7aTnaaBaaaleaacaWGQbaabeaakiabg2da9iaacIcadaaeqaqaaiaadIhadaWgaaWcbaGaamyAaiaadQgaaeqaaOGaaiykaaWcbaGaamyAaaqab0GaeyyeIuoakiaac+cacaGGJaGaam4zaiaadwgacaWGUbGaamyzaiaadohaaaa@6DF7@.

To discriminate between expression from two libraries, we use the work of Audic and Claverie [45], in which the probability of observing *y *reads from a library size of *N*_2 _reads, given that *x *reads are found from another library of size *N*_1_, is derived to yield the following:

p(y|x)=(N2N1)y(x+y)!x!y!(1+N2/N1)x+y+1
 MathType@MTEF@5@5@+=feaafiart1ev1aaatCvAUfeBSjuyZL2yd9gzLbvyNv2Caerbhv2BYDwAHbqedmvETj2BSbqee0evGueE0jxyaibaiKI8=vI8tuQ8FMI8Gi=hEeeu0xXdbba9frFj0=OqFfea0dXdd9vqai=hGuQ8kuc9pgc9s8qqaq=dirpe0xb9q8qiLsFr0=vr0=vr0dc8meaabaqaciGacaGaaeqabaqadeqadaaakeaacaWGWbGaaiikaiaadMhacaGG8bGaamiEaiaacMcacqGH9aqpdaqadaqaamaalaaabaGaamOtamaaBaaaleaacaaIYaaabeaaaOqaaiaad6eadaWgaaWcbaGaaGymaaqabaaaaaGccaGLOaGaayzkaaWaaWbaaSqabeaacaWG5baaaOWaaSaaaeaacaGGOaGaamiEaiabgUcaRiaadMhacaGGPaGaaiyiaaqaaiaadIhacaGGHaGaamyEaiaacgcacaGGOaGaaGymaiabgUcaRiaad6eadaWgaaWcbaGaaGOmaaqabaGccaGGVaGaamOtamaaBaaaleaacaaIXaaabeaakiaacMcadaahaaWcbeqaaiaadIhacqGHRaWkcaWG5bGaey4kaSIaaGymaaaaaaaaaa@53DC@

### Comparisons with Gene Ontology

To obtain GO [57] information for selected contigs of match levels M0 to M3 to UniProt, the GO annotation [47] was downloaded and fed into an SQL database, which allowed easy extraction of the data. We worked with the top level within each of the three main categories 'biological process', 'cellular component', and 'molecular function'. For each of these main categories, GO expression profiles were constructed as follows. The expressions of each of the top categories of 'molecular function' ('biological process' and 'cellular component') were accumulated and normalized into fractions. A pie chart of frequencies for each library was made and shown in Additional data files 2 to 4. We also computed an average pie chart for 'molecular function' ('biological process' and 'cellular component' respectively) and used it as the expected portion of a randomly chosen category. For each library we computed log odds ratios of the GO categories between the actual pie and the average pie. The log odds values for each main category in 'molecular function' ('biological process' and 'cellular component') were also considered for libraries grouped into corresponding tissues and over-representation of functional features, in accordance with the tissue functions identified.

### Online access to the resource

The resource described [29] contains a backend Distiller SQL server that can be accessed for retrieval of information on each contig. Furthermore, static data are available, such as fasta files of the contigs and singletons, BLAST to UniProt and a raw SNP list, as well as the presented expression values.

## Additional data files

The following additional data are available with the online version of this paper. Additional data file 1 contains figures related to the expression analysis, cleaning, and correlations; tables of noncoding RNA, house-keeping genes, and the top 50 cDNA-library-specific genes; and a table of the NCBI trace archive accession numbers. Additional data file 2 contains pie charts of how the expression for each cDNA library is distributed in the main GO category of 'molecular function'. Additional data file 3 contains pie charts of how the expression for each cDNA library is distributed in the GO category of 'cellular component'. Additional data file 4 contains pie charts of how the expression for each cDNA library is distributed in the GO category of 'biological process'.

## Supplementary Material

Additional data file 1 Additional data file 1 contains figures related to the expression analysis, cleaning and correlations. Furthermore, Additional data file 1 contains tables of noncoding RNA, housekeeping genes and the top 50 cDNA-library-specific genes. Finally, it contains a table of the NCBI trace archive accession numbers. Click here for file

Additional data file 2 Additional data file 2 contains pie charts of how the expression for each cDNA library is distributed in the main level of GO category 'molecular function'. Click here for file

Additional data file 3 Additional data file 3 contains pie charts of how the expression for each cDNA library is distributed in the GO category of 'cellular component'. Click here for file

Additional data file 4 Additional data file 4 contains pie charts of how the expression for each cDNA library is distributed in the GO category of 'biological process'. Click here for file

## Figures and Tables

**Table 1:** cDNA libraries

Lib name	Tissue (Animals)	Description	Reads
Amn	Amnion (S)	-	2,394
Aor	Aorta (M)	-	5,121
Bla	Bladder (M)	-	8,042
Nbm^a^	Bone marrow (S)	115 days, bone marrow	10,068
Cbe	Brain (M)	Cerebellum	4,180
Cbr^b^	Brain (B)	Brain (cortex)	7,814
Fco	Brain (M)	Frontal cortex	6,361
Hyp	Brain (S)	Hypothalamus	7,001
Pgl	Brain (M)	Pituitary gland	8,440
Ecc^a^	Brain (S)	F 50 days, cortex	8,693
Ece^a^	Brain (S)	F 50 days, cerebellum	4,361
Fce^a^	Brain (S)	F 100, cerebellum	3,682
Fcc^a^	Brain (S)	F 107, cortex cerebri	5,056
Fhi^a^	Brain (S)	F 107 Hippocampus	5,897
Cbl^b^	Haemopoetic (B)	Blood	8,711
Jca	Cartillage (S)	Joint capsule	8,775
Nca^a^	Cartillage (S)	115 days, cartilage	7,306
Pan^b^	Endocrine glands (M)	Pancreas	4,238
Ret	Eye (M)	Retina	7,768
Eye^a^	Eye (S)	F 50, eye	5,865
Fat	Fat (M)	Fat	6,783
Che	Heart (B)	-	7,336
Hea	Heart (M)	-	4,890
Hlv	Heart (S)	Left ventricle	7,181
Cje	Intestine (B)	Jejunum	6,052
Col	Intestine (S)	Large intest, colon asc.	5,128
Duo	Intestine (S)	Small intest, duodenum	5,787
Ill	Intestine (S)	Small intest, illeum	5,695
Jej	Intestine (S)	Small intest, jejunum	10,109
Lin	Intestine (M)	Large intestine	6,868
Sin	Intestine (M)	Small intestine	5,716
Eje^a^	Intestine (S)	F 50, Jejunum	10,118
Nco^a^	Intestine (S)	115 days, colon	6,183
Nje^a^	Intestine (S)	115 days, jejunum	6,027
Cki	Kidney (B)	-	6,052
Kid	Kidney (M)	-	7,708
Cli	Liver (B)	-	6,544
Liv	Liver (M)	-	6,836
Eli^a^	Liver (S)	F 50, liver	6,587
Fli^a^	Liver (S)	F 100, liver	4,929
Clu	Lung (B)	-	8,358
Lun^c^	Lung (M)	-	6,645
Elu^a^	Lung (S)	F 50 days, lung	2,595
Nlu^a^	Lung (S)	115 days, lung	5,217
Cly	Lymphatic gland (B)	-	8,289
Lyg	Lymphatic gland (M)	-	7,513
Lnt	Lymphatic gland (S)	-	7,027
Cga	Mammary gland (B)	-	3,583
Mcp	Mammary gland (S)	Mammae, collostrum prod	5,860
Mga	Mammary gland (M)	7 days after weaning	6,242
Mgm^b^	Mammary gland (M)	14 days after birth	5,545
Mgp	Mammary gland (M)	7 days pre-birth	4,335
Med	Mediastinum (S)	-	8,602
Bfe	Muscles (M)	M. biceps femoris	6,673
Ctl^b^	Muscles (B)	Tenderloin	6,533
Isp	Muscles (M)	M. infraspinatus	6,650
Ldo	Muscles (M)	M. longissimus dorsi	10,309
Mas	Muscles (S)	M. masseter	4,755
Sme	Muscles (M)	M. semimembranosus	3,274
Ssp	Muscles (M)	M. supraspinatus	7,379
Ste	Muscles (M)	M. semitendinosus	7,396
Tbr	Muscles (M)	M. triceps brachii	6,486
Vin	Muscles (M)	M. vastus intermedius	3,007
Ese^a^	Muscles (S)	F 50, M. semitendinosus	7,905
Nms^a^	Muscles (S)	115 days, M. semitendinosus	4,676
Gul	Oesophagus (M)	-	5,631
Ova	Ovary (M)	-	7,744
Cov	Ovary (S)	-	7,567
Pla^d^	Placenta (M)	-	7,481
Pro	Prostata (M)	-	1,953
Rec	Rectum (M)	-	5,778
Cmu	Rhinal mucosal membrane (B)	-	5,365
Nmm^a^	Rhinal mucosal membrane (S)	115 days, mucosal memb.	7,530
Sag	Salivary gland (M)	-	5,473
Csk	Skin (B)	-	7,105
Ski	Skin (M)	-	6,815
Ton	Skin (S)	Tip of tongue, mucosa	5,698
Eep^a^	Skin (S)	F 50, epidermis	8,159
Eru^a^	Skin (S)	F 50, regium bilicalis	8,330
Nep^a^	Skin (S)	115 days, epidermis	5,437
Spc	Spinal cord (M)	Spinal cord	8,821
Ebs^a^	Spinal cord (S)	F 50 days, brainstem	8,453
Fbs^a^	Spinal cord (S)	F 107 brainstem	5,703
Spl	Spleen (M)	-	6,984
Csp	Spleen (B)	-	6,204
Cst	Stomach (B)	-	7,141
Sto	Stomach (M)	-	5,561
Sug	Suprarenal glands (M)	-	7,856
Cag	Suprarenal glands (B)	Adrenal gland	6,614
Cte	Testicle (B)	-	3,416
Tes	Testicle (M)	-	4,812
Cty	Thyroid glands (B)	-	9,608
Thg	Thyroid glands (M)	-	7,887
Pty	Thyroid glands (S)	Piglet 2 days, thymus	7,007
Fty^a^	Thyroid glands (S)	F 100, thymus	5,687
Tra	Trachea (M)	-	8,124
Ute	Uterus (S)	-	7,531
Cut	Uterus (B)	-	5,885

The generated cDNA libraries, representing 35 tissues. They are here shown as two (overlapping) sets: a physiologic set and a developmental set. The column 'Lib name' gives three letter code for the library. 'Tissue' indicates the overall tissue the library was generated from, where '(Animals)' indicates whether the library was generated from a single (S or B) or multiple (M) animals. Libraries listed with (M) and (S) represent the pig breeds (mostly cross-breeds) used in Danish breeding (Landrace, Yorkshire, Duroc, and Hampshire), whereas the libraries listed with (B) present Chinese pig breeds. 'Description' provides a short description. The column 'Reads' shows the number of reads that went into that library after cleaning. The sum of all 'Reads' corresponds to the number of generated reads that contributed to the assembly, that is the number of reads after cleaning vector, repeats, and so on. Library names beginning with 'C' originates from Chinese pig breeds (except for 'Col' and 'Cbe'), whereas the remaining libraries originated from Danish pig breeds. ^a^Developmental tissue. ^b^Ignored in expression analysis (see Materials and methods). ^c^Likely to be heavily contaminated by liver expressed sequence tags. ^d^A normalized library.

**Table 2:** Match of contigs and singletons to known databases

Match level (ID/Sbj)	Contigs		Singletons	
	
	UniProt	NcRNAdb	UniProt	NcRNAdb
M0 (98%/100%)	1,982	21	173	6
M1 (95%/95%)	1,304	18	101	12
M2 (85%/90%)	2,517	72	236	20
M3 (70%/70%)	3,480	-	749	-
M4 (60%/50%)	3,603	-	1,355	-
M5 (20%/20%)	11,973	-	12,337	-

The table list the number of hits to given databases with various levels of matching for clusters and singletons. The cutoffs for given match level are indicated in terms of alignment identity (ID) and subject coverage (Sbj) for UniProt and the noncoding RNA databases (ncRNAdb). Only match levels up to M2 (alignment length larger than 30 nucleotides) for ncRNAs are included (counting each contig/singleton only once) and the matches have been cleaned for tRNAs because these appears to be the most frequent RNAs from contamination, such as *E. coli*. A curated list of ncRNAs for levels M0 and M1 can found in Additional data file 1 (Table S1). Also see text for details. It should be noted that a few conreads match the same UniProt ID. This can be due to phylogenetic decomposition or single reads not being assembled. The total number of contigs was 48,629; the number of singletons was 73,171.

**Table 3:** Primers and PCR conditions

PigEST name	Gene symbol	Oligo sequence (5' to 3')	Amplicon length	Tm (°C)
Ss1.1-rhlv24b_a21.5	Troponin	CCAGAGTCCCCAGGATA TGAGCAGGGCTTTATTTGCATTC	100	63
Ss1.1-rcst01_n6.5	Pepsinogen A precursor	TACTGCTGCTCAGCTTG GTGTCCTTCAGCTTGCCATTCTT	106	60
Ss1.1-rcst21_l12.5	Pepsinogen C	TCCTGGTCCTTTTTGACACCTAGAGGACTTGCTGGGGTTG	108	60
Ss1.1-rhyp08c_e13.5.5	Myelin basic protein	GCAGGGCATAGAGATGGTGTCCCGACCCTGTTAGGAAGAT	100	60
Ss1.1-Liv1-LVRM1E040203.5.5	Fetuin B	GCCCTGTGTTTCAAATCCTGAGGAGCCACAAGGACAGCTA	100	60
Ss1.1-rnlu1830b_g11.5	SP-C	TGTACATCTAGGAAACATCAGATTCTTTGGTGGTAGAAGCC	201	60
Ss1.1-rill310b_f20.5	Gastrotropin	TGAACAGCCCCAACTACCACTCATGCCAGCTTCTTGCTTA	110	60
Ss1.1-rduo424b_g21.5	Vitamin D-dependent calcium binding protein	TGAGTGCCCAAAAGTCTCCTCAGTTGCTTCAGCTCCTCCT	153	60

Table of the used primers and their corresponding polymerase chain reaction (PCR) conditions for the eight selected genes for which quantitative PCR (qPCR) was carried out. The column 'PigEST name' indicates the name of contig. 'Gene name' is the gene name of the selected genes according to UniProt match. 'Oligo sequence (5' to 3')' is the oligonucleotide sequences used in the qPCR experiment. 'Amplicon length' is the length of the amplified product in the qPCR. 'Tm' indicates the annealing temperature used in the qPCR.
