# Supplementary material for: Porcine transcriptome analysis based on 97 non-normalized cDNA libraries and assembly of 1,021,891 expressed sequence tags
Source: Genome Biol. 2007 Apr 2;8(4):R45. doi: 10.1186/gb-2007-8-4-r45 (PMC1895994; doi:10.1186/gb-2007-8-4-r45)
Supplement: Additional data file 1 — contains figures related to the expression analysis, cleaning and correlations. Furthermore, Additional data file 1 contains tables of noncoding RNA, housekeeping genes and the top 50 cDNA-library-specific genes. Finally, it contains a table of the NCBI trace archive accession numbers. [file gb-2007-8-4-r45-S1.pdf]

Supplementary material:

Porcine transcriptome analysis based on 97 non-normalized cDNA libraries and assembly of 1,021,891 ESTs

Jan Gorodkin[1], Susanna Cirera[1], Jakob Hedegaard[2], Michael J. Gilchrist[3], Frank Panitz[2], Claus B. Jørgensen[1], Karsten Scheibye-Knudsen[1], Troels Arvin[1], Steen Lumholdt[1], Milena Sawera[1], Trine Green[1], Bente J. Nielsen[1], Jakob H. Havgaard[1], Carina Rosenkilde[1], Jun Wang[4,5,6], Heng Li[4,5], Ruiqiang Li[4,6], Bin Liu[4], Songnian Hu[4], Wei Dong[4], Wei Li[4], Jun Yu[4], Jian Wang[4], Hans-Henrik Stærfeldt[7], Rasmus Wernersson[7], Lone B. Madsen[2], B. Thomsen[2], Henrik Hornshøj[2], B. Zahn[2], X. Wang[2], Lars Bolund[4,5], Søren Brunak[7], Huanming Yang[4], Christian Bendixen[2] Merete Fredholm[1*]

[1]Division of Genetics and Bioinformatics, IBHV The Royal Veterinary and Agricultural University, DK-1870 Frederiksberg C, Denmark

[2] Department of Genetics and Biotechnology, Danish Institute of Agricultural Sciences, DK-8830 Tjele, Denmark

[3] The Wellcome Trust/Cancer Research UK Gurdon Institute, Cambridge, CB2 1QN, UK

[4] Beijing Genomics Institute, Beijing 101300, P.R. China

[5] Institute of Human Genetics, University of Aarhus, DK-8000 Aarhus C, Denmark

[6] Department of Biochemistry and Molecular Biology, University of Southern Denmark, DK-5230 Odense M, Denmark

[7] Center for Biological Sequence Analysis, BioCentrum-DTU, Building 208, DK-2800 Lyngby, Denmark

January 17, 2007

Running title: A draft of the porcine transcriptome.

1 Cleaning libraries

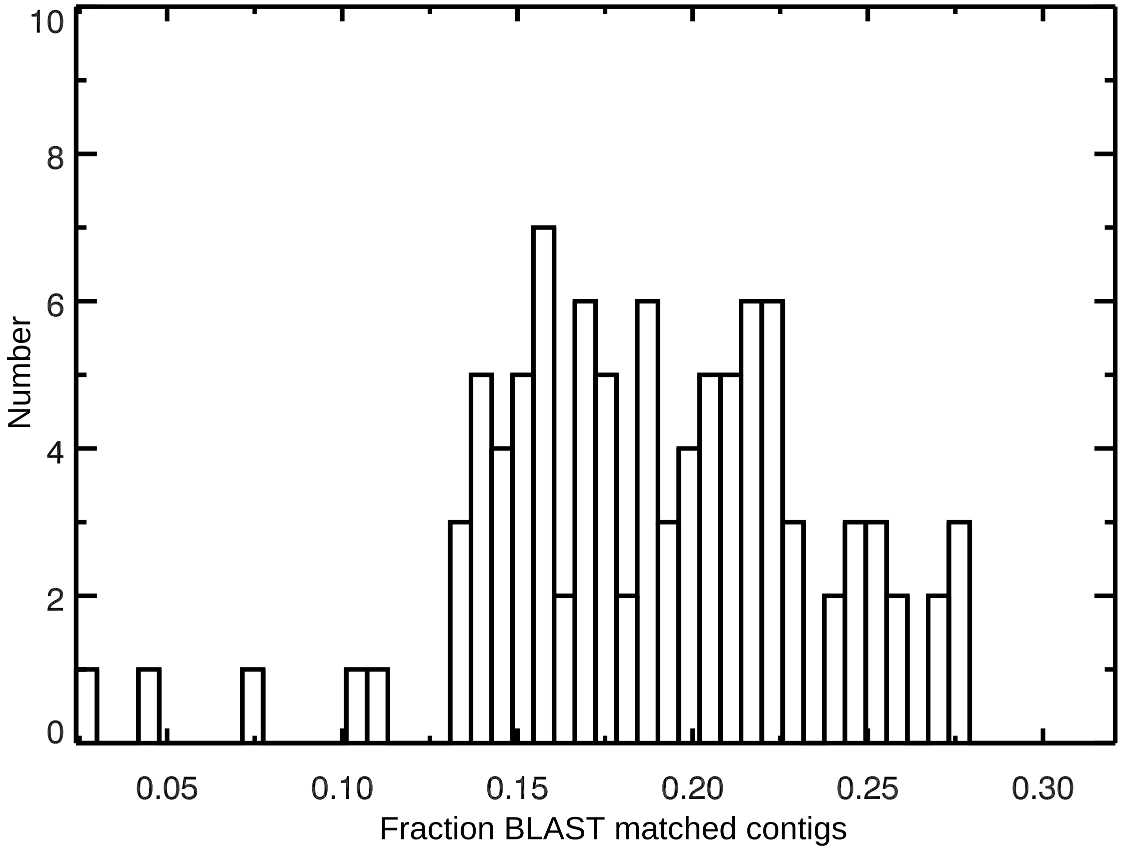

Figure S1: The distribution of the fraction of contigs with BLAST match of at least level M3]. There is a tail of fraction less than 12.5 % consisting of the 5 libraries: ctl, cbr, pan, cbl and mgm.

2 Patterns of expression correlation

Figure S2: Pairwise correlation of expression. (A) An example of correlating (CC: 0.81) expression between two ribosomal proteins homologous to RLA0_HUMAN and RS23_HUMAN. Each point in the plot represent a cDNA library and its coordinate the expression values of the two genes for that library. (B) An example of expression correlation (CC: 0.89) between two non-normalized developmental brain cDNA libraries, Fbs (foetus 107 days, brain stem) and Fhi (foetus 107 days, hippocampus). Each point in the plot represent a gene cluster and its coordinate the expression of the that in the two libraries.

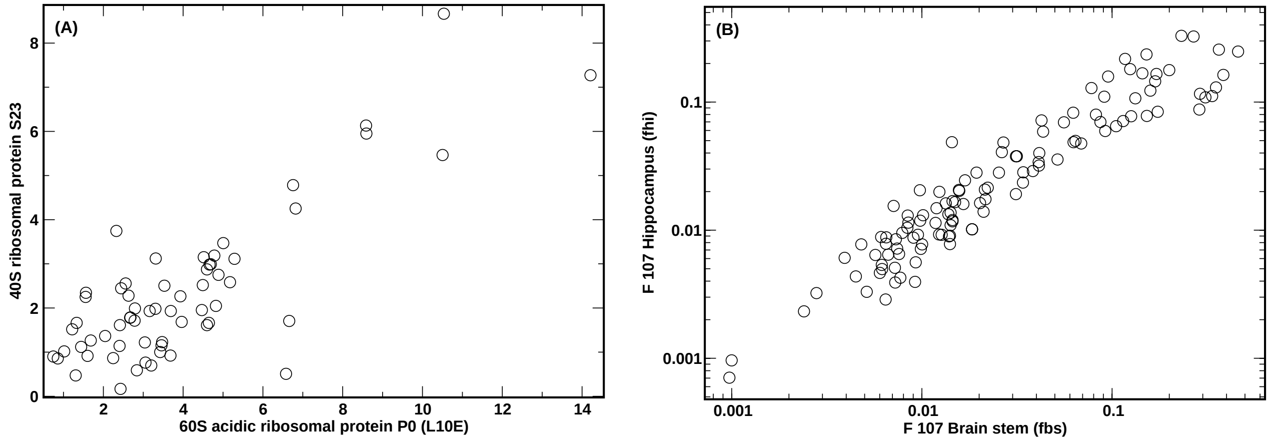

3 Patterns of expression correlation

Figure S3: An example of correlation between gene ontology categories across the tissues, here between *transcription regulator activity* and *translation regulator activity*. Each point represent the overall proportion of that library representation of the two categories.

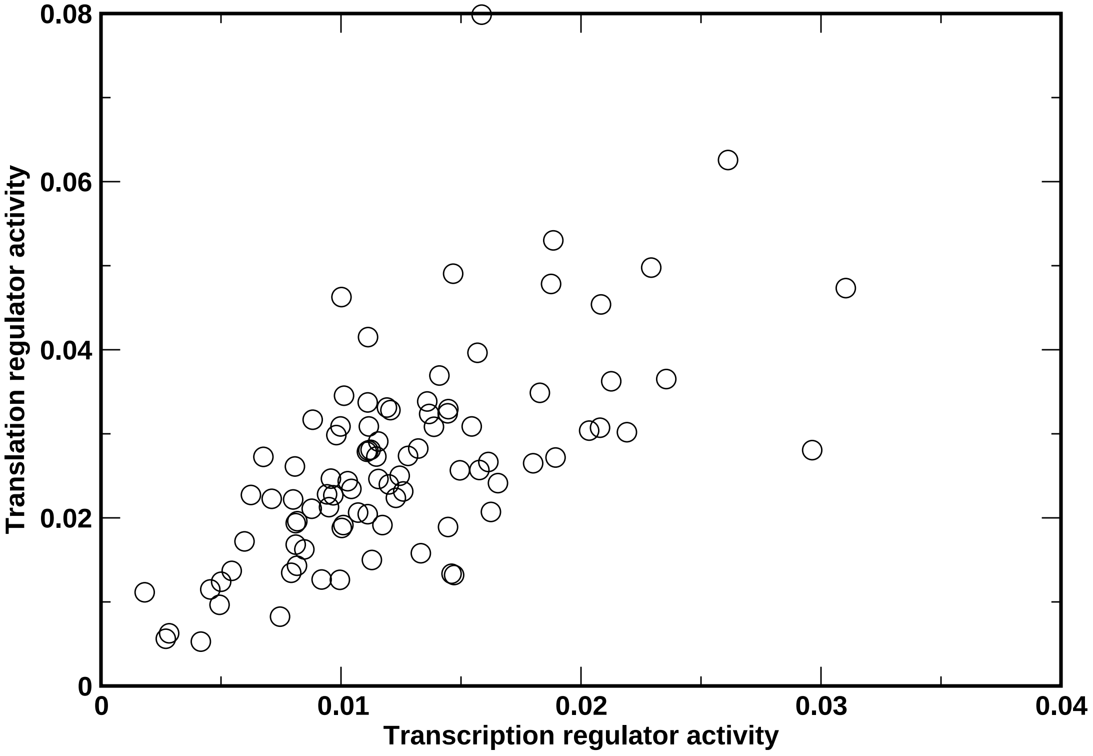

4 Non-coding RNA candidates

Table S1: The 53 non-coding RNA candidates found by similarity of matches M0 or M1 (see Table 2 in the main text). The column "SD sequence name" indicates the sequence name of the gene-cluster or singleton, "Seq. ID" is the sequence ID from various sources(*), "Description" provides a short description, "Rfam AC" the Rfam accession name (if available) and "Length" is the sequence length of the subject non-coding RNA from the database. (*) Names containing a "" are from Rfam, names containing "-mir" from the microRNA registry and names with a "|" originates from fantom or RNAdb. Notes: The following groups of clusters were only counted once for the different reasons: [1,3]: These sequences/clusters were not merged due to lack of overlap beyond the region matching the RNA. [2]: These clusters were the result of phylogenetic decomposition from an original cluster, thus seperating the EST from different strains. Furthermore two singletons Tra1-TCH01E100031 and Pig1-2O15 were discarded as the match bacterial RNAs.

SD sequence name	Seq. ID	Description	Rfam AC	Length
[1]Ss1.1-Pig3-SRG8017G22.5	X00722.1/165-317	5_8S_rRNA 5.8S ribosomal RNA	RF00002	149
[1]Ss1.1-Liv1-LVRM10156E03.5	X00722.1/165-317	5_8S_rRNA 5.8S ribosomal RNA	RF00002	153
Ss1.1-rcad24b_l22.5	AC025362.12/104594-104663	snoZ12 small nucleolar RNA	RF00289	67
rpigca_2218.y1	AF146043.1/3911-3989	snoZ37 Small nucleolar RNA	RF00055	79
Pig1-121B20	AL357314.11/74156-74233	U45 small nucleolar RNA	RF00279	78
Ss1.1-rret20_p23.5	AB070559.1/4340-4471<br>gnl|combinedlit|LIT1477	U72 small nucleolar RNA	RF00139	132
Ss1.1-MPL-MPL384_4_A01.5	AC112907.7/62238-62107<br>gnl|combinedlit|LIT1126	E3 small nucleolar RNA	RF00092	133
Ss1.1-LyN1-MLN01A050104.5	AF532927.1/678-740	SECIS Selenocysteine insertion sequence	RF00031	63
Ss1.1-LyN1-MLN01A060101.5	AY358096.1/701-767	SECIS Selenocysteine insertion sequence	RF00031	67
Ss1.1-ruio20_b4.5	D88033.1/3186-3249	SECIS Selenocysteine insertion sequence	RF00031	63
Ss1.1-Liv1-LVR010052B09.5	L12743.1/694-758	SECIS Selenocysteine insertion sequence	RF00031	65
Ss1.1-recc2834c_f22.5	AF380118.1/366-433	SECIS Selenocysteine insertion sequence	RF00031	68
[2]Ss1.1-Tra1-TCH01E080083.5.120	M11778.1/1726-1789	SECIS Selenocysteine insertion sequence	RF00031	64
[2]Ss1.1-Ova1-OVR010002E09.5.110	M11778.1/1726-1789	SECIS Selenocysteine insertion sequence	RF00031	64
Bra1-olf_141_F03	AK022146.1/1-191	U2 spliceosomal RNA	RF00004	190
Bra1-olf_091_B07	U57614.1/4882-5072	U2 spliceosomal RNA	RF00004	186
rtes07_n1.y1	BX648500.1/2-97	U6 spliceosomal RNA	RF00026	93
[3]rnco1919b_l17.y1	AK127657.1/1-85	U6 spliceosomal RNA	RF00026	83
[3]Pig1-35H07	AK127657.1/1-85	U6 spliceosomal RNA	RF00026	83
[3]Liv1-LVR01C070083	AK127657.1/1-85	U6 spliceosomal RNA	RF00026	81
Pla1-C0000031_K16	AC131676.2/83760-83866	U6 spliceosomal RNA	RF00026	107
Ss1.1-Pig4-TMW8034N20.5	M31687.1/705-811	U6 spliceosomal RNA	RF00026	107
Ss1.1-Pig3-SRG8023E21.5	AL806519.12/25496-25390	U6 spliceosomal RNA	RF00026	107
reep15c_p11.y1	rno-mir-365	pre-miRNA	—	86
rplac_6675.y1	mmu-mir-425	pre-miRNA	—	85
Pig1-58H11	ppa-mir-24-1<br>gnl|combinedlit|LIT3258<br>AF043896.1/53-120	pre-miRNA	RF00178	68
Pig2-36J18	hsa-mir-423	pre-miRNA	—	92
Ss1.1-rpigcb_15937.5	ssc-mir-145	pre-miRNA	—	86
Ss1.1-unkn-MI-P-NA-aea-d-09-1-UM.s1.ab1.3	ssc-mir-186	pre-miRNA	—	82
Ss1.1-Pig4-TMW8022F14.3	ssc-mir-214<br>gnl|combinedlit|LIT3238	pre-miRNA	—	110
Ss1.1-Pig2-80M18.5	hsa-mir-137<br>gnl|combinedlit|LIT2562	pre-miRNA	—	102
Ss1.1-Pig2-138B11.5.5	ssc-mir-29c<br>gnl|combinedlit|LIT2927	pre-miRNA	—	88
Ss1.1-rpldo0104_e22.5	rno-mir-133a<br>gnl|combinedlit|LIT2505<br>AC105947.11/187382-187294	pre-miRNA	RF00446	87
Ss1.1-OvaP-UMC-pputal4-001-a11.3	hsa-let-7b<br>gnl|combinedlit|LIT2458<br>AL049853.1/17070-16986	pre-miRNA	RF00027	84
rnmm27c_f4.y1	AF026006.1/1132-1245	K_chan_RES Potassium channel RNA editing signal	RF00485	111
Ss1.1-Pig2-65E16.5	AC079587.4/53411-53524	K_chan_RES Potassium channel RNA editing signal	RF00485	109
Ss1.1-Pig3-SRG8005M11.5	AC006063.1/55115-55002	K_chan_RES Potassium channel RNA editing signal	RF00485	114
Ss1.1-Pig2-130P20.5	AF493545.1/787-900	K_chan_RES Potassium channel RNA editing signal	RF00485	114
Ss1.1-LyN1-MLN01H080062.5.5	AF258463.1/230-288	Antizyme_FSE Antizyme RNA<br>frameshifting stimulation element	RF00381	59
Ss1.1-Pig4-TMW8041I04.3	AF057297.1/248-303	Antizyme_FSE Antizyme RNA<br>frameshifting stimulation element	RF00381	56
Thy1-THY010037H12	X96401.1/13-215	IRES_mnt Mnt IRES	RF00457	199
Ss1.1-rpgl07_c11.5	AY196206.1/633-564	RNA-OUT RNA-OUT	RF00240	70
Ss1.1-Pig3-SRG8020F17.3	AF221942.1/106-459	Telomerase-vert Vertebrate<br>telomerase RNA	RF00024	355
Ss1.1-unkn-F_2_A09.5	U68481.1/913-1007	G-CSF_SLDE G-CSF factor stem-loop<br>destabilising element (SLDE)	RF00183	95
Ss1.1-rfat0118_g1.5	U70060.1/1239-1321	CAESAR ctgf/hcs24 CAESAR	RF00172	84
Ss1.1-Spl1-SPL010010E02.5	AY600299.1/4-151	Spi-1 (PU.1) 5' UTR regulatory element	RF00232	148
Ovi2-UMC-peov1-003-e07	ri|2900060B14|ZX00069H11|122	—	—	123
Ova2-C0003260_N12	ri|2010105B14|ZX00081B13|330	—	—	324
Ss1.1-Tra1-TCH01E080001.5	gnl|hinv|HIV2680	—	—	744
Ss1.1-Lng1-LNG010027D07.5	gnl|overlapseqs|ASO2290	—	—	152
Ss1.1-rfty538b_b18.5	ri|2700074F11|ZX00160O08|458	—	—	453
Ss1.1-rste35_j22.5	ri|2210404A05|ZX00051N24|201	—	—	201
Ss1.1-Pey1-15B06.5	gnl|fantom2|FT21316<br>ri|1110058D08|R000018D02|528	—	—	524
Ss1.1-Pig4-TMW8062O08.5	ri|1500006B23|ZX00050A13|105	—	—	104
Ss1.1-rspc032_g9.5	ri|2810470K03|ZX00067H11|224	—	—	225
Ss1.1-Pig4-TMW8036O13.3	ri|A730009J10|PX00724L22|429	—	—	428
Ss1.1-Pig4-TMW8039D24.3	ri|E430019M05|PX00099H09|851	—	—	830

5 House keeping gene candidates

Table S2: The gene-clusters (next page) represented in more than 80 libraries. The column "SD sequence name" indicates the sequence name of the gene-cluster, the column "E-level" the average (row) unnormalized expression, which is the expected fraction ($\times$ 1000) of that gene in a library, "M-level" is the match level to uniprot and "Description" is the description form the matching sequence in uniprot.

SD sequence name	E-level	#Libs	M-level	Description
Ss1.1-LyN1-MLN01F050053.5.5	15.6678	92	M0	TCTP_PIG (P61288) Translationally controlled tumor protein (TCTP)
Ss1.1-Lng1-LNG010078H09.5.5	3.3581	92	M0	RLA0_HUMAN (P05388) 60S acidic ribosomal protein P0 (L10E)
Ss1.1-Liv1-LVR010102H02.5.5	7.7001	92	M0	Q6IPS9_HUMAN (Q6IPS9) Eukaryotic translation elongation factor 1 alpha 1 (MSTP056)
Ss1.1-Tes1-TES01A020049.5	1.6710	91	M0	Q545X8_MOUSE (Q545X8) Adult male kidney cDNA, RIKEN full-length enriched library, clone:0610010B10 product:ribosomal protein S4, X-linked, full insert sequence (ES cells cDNA, RIKEN full-length enriched library, clone:2410045N08 product:ribosomal protein
Ss1.1-rtra07_p8.5.5	2.1168	91	M1	RL6_BOVIN (Q58DQ3) 60S ribosomal protein L6
Ss1.1-rsug26_p8.5.5	2.8581	91	M0	Q5BLK1_MOUSE (Q5BLK1) Ribosomal protein S6
Ss1.1-rmcp35c_c12.5	3.4855	91	M0	Q6GMC1_XENLA (Q6GMC1) MGC81889 protein
Ss1.1-rlyg16_g3.5.5	6.2989	91	M0	Q6ZWX2_MOUSE (Q6ZWX2) Mus musculus ES cells cDNA, RIKEN full-length enriched library, clone:2410042N11 product:thymosin, beta 4, X chromosome, full insert sequence (Thymosin, beta 4, X chromosome)
Ss1.1-LyN1-MLN01E070009.5.5	1.7872	91	M0	Q9H3F4_HUMAN (Q9H3F4) MSTP030 (Ribosomal protein L5)
Ss1.1-Utr1-UTR010031E07.5	1.9760	90	M0	PTMA_BOVIN (P01252) Prothymosin alpha
Ss1.1-rspc005_k12.5.5	1.4393	90	M0	Q14433_HUMAN (Q14433) Guanine nucleotide-binding protein G-s-alpha-3; NCBI gi: 386745 (OTTHUMP00000064258) (Fragment)
Ss1.1-rpgl23_j16.5.5	1.9881	90	M0	Q6PHZ1_MOUSE (Q6PHZ1) Rpl17 protein
Ss1.1-rfat0140_c19.5	3.0357	90	M0	Q53Z07_HUMAN (Q53Z07) Ribosomal protein L9 isoform (NPC-A-16)
Ss1.1-rpgl25_m6.5.5	1.3753	89	M0	Q5E983_BOVIN (Q5E983) Eukaryotic translation elongation factor 1 beta 2
Ss1.1-rjej35b_g5.5.5	2.5156	89	M0	Q642L0_MOUSE (Q642L0) Ribosomal protein S24, isoform 1
Ss1.1-rebs14c_l6.5	2.1297	89	M0	Q5VVD0_HUMAN (Q5VVD0) Ribosomal protein L11
Ss1.1-rnco1922b_f18.5	1.3337	88	M0	Q6IAX2_HUMAN (Q6IAX2) RPL21 protein (Ribosomal protein L21)
Ss1.1-rjej40b_g8.5.5	3.2764	88	M0	Q91YK6_MOUSE (Q91YK6) Rpl23a protein (Fragment)
Ss1.1-rebs30c_o18.5.5	1.6066	88	M0	Q63ZV8_RAT (Q63ZV8) Ribosomal protein L32
Ss1.1-Lng1-LNG010013F05.5	1.1753	88	M0	Q6PI79_XENLA (Q6PI79) MGC52708 protein (MGC78769 protein) (H3f3b-prov protein) (H3f3a-prov protein) (MGC81913 protein)
Ss1.1-Lng1-LNG010002A09.5	1.8477	88	M2	Q59GY2_HUMAN (Q59GY2) Ribosomal protein L4 variant (Fragment)
Ss1.1-Liv1-LVR010055D02.5	1.2714	88	M0	Q5RA83_PONPY (Q5RA83) Hypothetical protein DKFZp459D1928
Ss1.1-Utr1-UTR010020F01.5.5	3.9889	87	M0	B2MG_PIG (Q07717) Beta-2-microglobulin precursor (Lactollin)
Ss1.1-Tra1-TCH01A030070.5	1.4871	87	M0	Q52N47_PIG (Q52N47) Integral membrane protein 2B
Ss1.1-rlnt31c_g18.5.5	3.3976	87	M0	Q6ZWZ6_MOUSE (Q6ZWZ6) Mus musculus adult male kidney cDNA, RIKEN full-length enriched library, clone:0610039M20 product:ribosomal protein S12, full insert sequence (Ribosomal protein S12) (Mus musculus adult male small intestine cDNA, RIKEN full-length e
Ss1.1-reru05c_o5.5	2.4411	87	M0	Q5BLK2_MOUSE (Q5BLK2) Ribosomal protein S20
Ss1.1-reje04b_g3.5	1.2151	87	M0	Q5M8Q0_MOUSE (Q5M8Q0) Ribosomal protein L15
Ss1.1-LyN1-MLN01H080062.5.5	1.0986	87	M1	Q29176_PIG (Q29176) Ornithine decarboxylase antizyme (Fragment)
Ss1.1-LyN1-MLN01F090002.5	1.0447	87	M0	Q6PI79_XENLA (Q6PI79) MGC52708 protein (MGC78769 protein) (H3f3b-prov protein) (H3f3a-prov protein) (MGC81913 protein)
Ss1.1-Tra1-TCH01H100090.5.5	1.6565	86	M0	Q6INP3_XENLA (Q6INP3) Hypothetical protein
Ss1.1-Tra1-TCH01D040102.5.5	2.7785	86	M0	TBAK_HUMAN (P68363) Tubulin alpha-ubiquitous chain (Alpha-tubulin ubiquitous) (Tubulin K-alpha-1)
Ss1.1-rspl17_f22.5	2.6075	86	M0	Q56JV9_BOVIN (Q56JV9) Ribosomal protein S3a
Ss1.1-rmed33c_f21.5	3.1659	86	M0	Q5E958_BOVIN (Q5E958) Ribosomal protein S8
Ss1.1-rhlv33b_e8.5.5	0.9905	86	M0	Q95282_PIG (Q95282) ATP synthase subunit 9, mitochondrial (Fragment)
Ss1.1-Utr1-UTR010044D06.5	2.2164	85	M0	Q548L2_HUMAN (Q548L2) CTCL tumor antigen HD-CL-06 (Vimentin variant)
Ss1.1-Tra1-TCH01H020010.5	0.9182	85	M2	Q5I0W0_MOUSE (Q5I0W0) Atp5f1 protein
Ss1.1-rnca08b_d7.5	0.7617	85	M2	CHCH2_HUMAN (Q9Y6H1) Coiled-coil-helix-coiled-coil-helix domain containing protein 2 (HCV NS2 trans-regulated protein) (NS2TP)
Ss1.1-reru34c_m15.5	1.1336	85	M0	Q58EA4_MOUSE (Q58EA4) Ribosomal protein L24
Ss1.1-rebs23c_c16.5	1.4328	85	M0	Q5BLJ7_MOUSE (Q5BLJ7) Ribosomal protein S13
Ss1.1-LyN1-MLN01F120059.5.5	1.4882	85	M0	Q95LX4_MACFA (Q95LX4) Hypothetical protein
Ss1.1-Tra1-TCH01B110046.5	0.9542	84	M0	ATPB_BOVIN (P00829) ATP synthase beta chain, mitochondrial precursor (EC 3.6.3.14)
Ss1.1-rpldo0136_n6.5	0.6539	84	M0	Q5E9A3_BOVIN (Q5E9A3) Poly(RC) binding protein 1
Ss1.1-rpgl03_d11.5	0.7218	84	M0	Q5U3Y8_RAT (Q5U3Y8) Basic transcription factor 3 (Predicted)
Ss1.1-rjca33c_n9.5	1.3651	84	M0	Q5XJF6_MOUSE (Q5XJF6) Ribosomal protein L10A
Ss1.1-rjca20c_e21.5.5	1.7946	84	M0	Q542K7_MOUSE (Q542K7) 2 days neonate thymus thymic cells cDNA, RIKEN full-length enriched library, clone:E430003K16 product:unclassifiable, full insert sequence (2 days neonate thymus thymic cells cDNA, RIKEN full-length enriched library, clone:C920005B2
Ss1.1-rfat0107_a23.5	1.3492	84	M0	Q6QAS5_PIG (Q6QAS5) 60S ribosomal protein L12 (Fragment)
Ss1.1-recc2833c_n13.5.5	1.1404	84	M0	Q13765_HUMAN (Q13765) Nascent polypeptide associated complex alpha subunit (Alpha NAC) (NAC alpha) (NACA protein)
Ss1.1-rnbm24b_j1.5	0.9093	83	M0	Q5R556_PONPY (Q5R556) Hypothetical protein DKFZp459G1118 (Hypothetical protein DKFZp459K1710)
Ss1.1-rnbm15b_p14.5	1.8502	83	M0	Q58DT1_BOVIN (Q58DT1) Ribosomal protein L7
Ss1.1-rmed17c_f9.5	1.1102	83	M0	Q53ZT4_PIG (Q53ZT4) Peptidyl-prolyl cis-trans isomerase A
Ss1.1-rese40c_e16.5.5	2.1359	83	M0	Q7M2Y2_BOVIN (Q7M2Y2) Tumor-specific transplantation antigen P198 homolog p23 (Fragment)
Ss1.1-LyN1-MLN01H080026.5.5	1.1617	83	M2	Q7Z7N7_HUMAN (Q7Z7N7) SLC25A3 protein (Fragment)
Ss1.1-rmgp001_l24.5	1.1443	82	M2	Q53YE6_HUMAN (Q53YE6) Insulin-like growth factor binding protein 7
Ss1.1-rmas932b_l11.5	3.3590	82	M0	Q56K14_BOVIN (Q56K14) Ribosomal protein P1 isoform 1-like
Ss1.1-rjej02b_k17.5	2.4719	82	M0	Q56K03_BOVIN (Q56K03) Ribosomal protein L27a-like
Ss1.1-reje08b_h24.5	2.0149	82	M0	Q57Z92_HUMAN (Q57Z92) Hypothetical protein RPS7
Ss1.1-recc2803c_e10.5.5	0.7475	82	M0	Q5T7C3_HUMAN (Q5T7C3) High-mobility group box 1
Ss1.1-recc2802c_c11.5	1.1792	82	M0	Q58EA6_MOUSE (Q58EA6) Ribosomal protein S25 (Hypothetical protein)
Ss1.1-LyN1-MLN01B030076.5	0.5965	82	M0	BI1_PIG (Q66RM2) Bax inhibitor-1 (BI-1) (Testis enhanced gene transcript)
Ss1.1-Utr1-UTR01B070092.5.5	0.9914	81	M0	Q5FVF0_HUMAN (Q5FVF0) Nuclease sensitive element binding protein 1
Ss1.1-rmed02c_i19.5	1.9613	81	M0	RS16_RAT (P62250) 40S ribosomal protein S16
Ss1.1-reje02b_b12.5	0.8470	81	M0	SODC_PIG (P04178) Superoxide dismutase [Cu-Zn] (EC 1.15.1.1)
Ss1.1-rduo434b_e14.5.5	0.7004	81	M0	Q91ZR9_MOUSE (Q91ZR9) Heterogeneous nuclear ribonucleoprotein A2/B1
Ss1.1-LyN1-MLN01D010060.5	0.7667	81	M0	Q56JZ5_BOVIN (Q56JZ5) Eukaryotic translation initiation factor 3 subunit 3 gamma
Ss1.1-Lng1-LNG010027C01.5	0.7831	81	M0	ATPA1_BOVIN (P19483) ATP synthase alpha chain heart isoform, mitochondrial precursor (EC 3.6.3.14)

6 cDNA library specific genes

Table S3: Top 50 of the tissue specific gene candidates, when ignoring the two contigs (Ss1.1-rcmu33_m2.5,Ss1.1-rssp31_a10.5) not having a unigene match at all as well as the read Ss1.1-rcsk_0162.5 which most likely seems to be a rice contaminated read. The complete list consist of 1002 candidates and is available online via http://pigest.kvl.dk/download. The column "SD sequence name" indicates the sequence name of the gene-cluster and the label in paranthesis indicates whether a similar expression pattern was found (+) not found (−) or not available (?) in the Unigene resource. The column "Lib" the library, "E-level" the (row) unnormalized expression ($\times$ 1000). The columns with "(2)" indicates the same information for the second most expressed library. "None" for Lib(2) means that there are no other libraries with expression and the #R(2) value is sat "-1". The column "M-level" is the match level to uniprot and "Description" is the description form the matching sequence in uniprot. The column "UG" indicates if similar expression pattern was found in UniGene ('+'), nor found ('–'), not enough data to decide ('?').

SD sequence name	Lib	E-level	#R	Lib(2)	E-level(2)	#R(2)	P-val	M-level	Description	UG
Ss1.1-rsag001_a21.5	sag	189.4756	1037	pty	1.9980	14	0.00000e+00	M5	Q8HY86_BOVIN (Q8HY86) Statherin	+
Ss1.1-rpro31_i2.5	pro	246.7998	482	tes	5.8188	28	1.76784e-219	M0	MSMB_PIG (O02826) Beta-microseminoprotein precursor (Prostate secreted seminal plasma protein) (Prostate secretory protein PSP94) (PSP-94) (TS507)	+
Ss1.1-rnmm34c_j5.5	nmm	115.0066	866	cmu	1.8639	10	3.02209e-184	M0	Q8WMH1_PIG (Q8WMH1) Odorant binding protein	+
Ss1.1-rfli704b_j16.5.5	fli	199.8377	985	eli	37.0427	244	7.50800e-159	M0	Q29014_PIG (Q29014) Alpha-1 acid glycoprotein (Fragment)	+
Ss1.1-Liv1-LVR010068E11.5.5	lun	125.0564	831	cli	12.2249	80	2.01617e-156	M0	ALBU_PIG (P08835) Serum albumin precursor	–
Ss1.1-rcst21_l12.5	cst	99.7059	712	sto	5.9342	33	2.30008e-133	M3	Q9GMY3_RHIFE (Q9GMY3) Pepsinogen C	+
Ss1.1-rcmu33_m2.5	cmu	60.2050	323	nmm	0.9296	7	7.37968e-112			?
Ss1.1-Tra1-TCH01F090076.5	pty	64.7924	454	nmm	4.3825	33	4.28277e-103	M0	Q5XW65_PIG (Q5XW65) Lung and nasal epithelium carcinoma associated protein precursor	+
Ss1.1-rduo424b_g21.5	duo	69.1204	400	cje	3.9656	24	2.12099e-93	M0	S100G_PIG (P02632) Vitamin D-dependent calcium-binding protein, intestinal (CABP) (Calbindin D9K)	+
Ss1.1-rpgl01_k14.5	pgl	112.3223	948	None	0.0000	-1	1.62870e-86	M0	GLHA_PIG (P01219) Glycoprotein hormones alpha chain precursor (Follitropin alpha chain) (Follicle-stimulating hormone alpha chain) (FSH-alpha) (Lutropin alpha chain) (Luteinizing hormone alpha chain) (LSH-alpha) (Thyrotropin alpha chain) (Thyroid-stimulating hormone alpha chain) (TSH-alpha)	?
Ss1.1-rnca15b_h14.5	nca	43.2521	316	nmm	1.8592	14	3.57334e-78	M5	O77753_CANFA (O77753) Type IIA procollagen	+
Ss1.1-LyN1-MLN01E050012.5	ute	39.5698	298	nmm	1.4608	11	2.50727e-74	M0	PPA5_PIG (P09889) Tartrate-resistant acid phosphatase type 5 precursor (EC 3.1.3.2) (TR- AP) (Tartrate-resistant acid ATPase) (TrATPase) (Acid phosphatase 5, tartrate resistant) (Uteroferrin) (UF)	?
Ss1.1-Mixn-0007g14.5	ton	37.9080	216	eep	2.2062	18	4.54147e-62	M3	Q6AZK5_HUMAN (Q6AZK5) KRT13 protein (Fragment)	+
Ss1.1-Liv1-LVR010082F10.5	lun	35.6659	237	eli	1.6700	11	1.30236e-56	M2	FIBB_BOVIN (P02676) Fibrinogen beta chain precursor [Contains: Fibrinopeptide B]	–
Ss1.1-rlyg03_k7.5.5	lyg	100.3594	754	pty	35.1077	246	7.41299e-54	M0	Q863D3_PIG (Q863D3) Pheromaxein A subunit precursor	–
Ss1.1-rcje20_p13.5	cje	64.6067	391	duo	12.0961	70	9.69001e-53	M0	FABPL_PIG (P49924) Fatty acid-binding protein, liver (L-FABP)	–
Ss1.1-rlyg07_l20.5	lyg	38.3336	288	pty	4.4241	31	3.47117e-50	M5	STAT_HUMAN (P02808) Statherin precursor	?
Ss1.1-rnca31b_e11.5.5	ski	96.5517	658	fat	33.6134	228	2.17113e-49	M1	CO1A2_CANFA (O46392) Collagen alpha 2(I) chain precursor	+
Ss1.1-Pig4-TMW8025O13.3	eye	21.8244	128	ret	0.1287	1	4.07931e-46	M0	Q95KK5_RABIT (Q95KK5) Beta A1-crystallin	+
Ss1.1-Mixn-0025a16.5	pro	34.8182	68	fat	0.1474	1	6.86818e-44	M5	Q6UW28_HUMAN (Q6UW28) HRPE773	–
Ss1.1-rpigca0_007150.5	clu	23.8095	199	fty	0.3517	2	2.71671e-42	M4	AOAH_HUMAN (P28039) Acyloxyacyl hydrolase precursor (EC 3.1.1.77)	–
Ss1.1-rton1207b_j1.5	ton	24.2190	138	eye	0.1705	1	1.33915e-41	M4	Q5T527_HUMAN (Q5T527) Small proline-rich protein 1A	+
Ss1.1-rnca05b_c15.5.5	ute	28.2831	213	spc	3.6277	32	3.00754e-41	M0	OSTP_PIG (P14287) Osteopontin precursor (Bone sialoprotein 1) (Secreted phosphoprotein 1) (SPP-1)	–
Ss1.1-rlyg29_p4.5	lyg	29.8150	224	pty	3.1397	22	6.07476e-41	M4	Q6P7U5_MOUSE (Q6P7U5) Muc19 protein (Fragment)	?
Ss1.1-rcst01_l10.5.5	sto	91.8899	511	cst	35.5692	254	2.90498e-38	M0	GKN1_PIG (Q8HYA9) Gastrokine 1 precursor (18 kDa antrum mucosa protein) (AMP-18)	+
Ss1.1-rpgl22_i13.5.5	pgl	47.3934	400	None	0.0000	-1	5.62612e-37	M0	PRL_PIG (P01238) Prolactin precursor (PRL)	+
Ss1.1-rpty604b_o4.5	pty	31.9680	224	nmm	5.0465	38	6.07771e-37	M5	Q98988_SALFO (Q98988) Ovulatory protein-2 precursor	–
Ss1.1-rpigca0_009159.5.5	clu	31.3472	262	liv	4.9737	34	4.33793e-36	M0	Q811L7_MOUSE (Q811L7) Heterogeneous nuclear ribonucleoprotein H1	–
Ss1.1-rnmm37c_n3.5	nmm	23.9044	180	pty	2.1407	15	3.40377e-35	M0	Q866S8_PIG (Q866S8) Von Ebner gland protein	+
Ss1.1-rill310b_f20.5	ill	24.0562	137	fty	0.8792	5	4.41206e-35	M0	ILBP_PIG (P10289) Gastrotropin (GT) (Ileal lipid-binding protein) (ILBP) (Porcine ileal peptide) (PIP)	+
Ss1.1-Utr2-C0001810_J21.5	ute	43.6861	329	cut	9.6856	57	6.08418e-35	M1	UFAP2_PIG (P46202) Uteroferrin-associated basic protein 2 precursor (UABP-2)	+
Ss1.1-Liv1-LVR010066B06.5	cli	40.9535	268	lun	8.7284	58	8.09752e-35	M0	HPT_PIG (Q8SPS7) Haptoglobin precursor	+
Ss1.1-Tra1-TCH01E080083.5.120	cov	31.4524	238	cki	4.6266	28	8.16804e-34	M0	Q6UJZ1_PIG (Q6UJZ1) Glutathione peroxidase 3 (Fragment)	–
Ss1.1-rsag04_g11.5	sag	19.0024	104	pty	0.4281	3	9.05359e-34	M4	Q6P5S2_HUMAN (Q6P5S2) Hypothetical LOC389429 (Chromosome 6 open reading frame 58)	?
Ss1.1-rcsk_0162.5	csk	41.3793	294	None	0.0000	-1	7.70674e-32	M4	Q84XR3_HORSP (Q84XR3) Truncated alcohol dehydrogenase	?
Ss1.1-rute2121b_l1.5.5	ute	19.7849	149	cut	0.8496	5	2.66072e-31	M5	Q28388_HORSE (Q28388) P19 lipocalin	?
Ss1.1-Liv1-LVR010061D12.5	lun	26.0346	173	fli	1.8259	9	2.66125e-31	M3	FIBG_HUMAN (P02679) Fibrinogen gamma chain precursor	–
Ss1.1-rcst01_n6.5	cst	33.3287	238	sto	5.5745	31	5.35977e-31	M0	Q29080_PIG (Q29080) Pepsinogen A precursor	+
Ss1.1-Liv1-LVR010001B06.5	cli	19.5599	128	clu	2.0340	17	2.47899e-29	M3	Q9GKP1_PIG (Q9GKP1) Complement component C3 (Complement C3)	+
Ss1.1-rfat0140_a13.5	fat	26.6844	181	ski	5.1357	35	8.95358e-26	M0	FABPA_PIG (O97788) Fatty acid-binding protein, adipocyte (AFABP) (Adipocyte lipid-binding protein) (ALBP) (A-FABP) (AP2)	+
Ss1.1-Utr2-C0001801_G11.5.5	ute	11.5523	87	eep	0.2451	2	9.39906e-26	M0	Q7YQE5_PIG (Q7YQE5) Placenta expressed transcript protein	+
Ss1.1-rcsk27_l4.5	csk	16.0450	114	nep	0.5518	3	8.78731e-25	M5	Q67V03_ORYSA (Q67V03) Hexose transporter-like protein	?
Ss1.1-rcga37b_b23.5	cga	20.0949	72	mgp	0.2307	1	2.90257e-24	M0	WAP_PIG (O46655) Whey acidic protein precursor (WAP)	?
Ss1.1-Liv1-LVR010021H02.5	ute	14.7391	111	clu	1.4358	12	3.12333e-24	M3	Q5R5T2_PONPY (Q5R5T2) Hypothetical protein DKFZp459H066	?
Ss1.1-rmga10_g19.5	cga	27.3514	98	mga	4.0051	25	4.20456e-23	M0	LALBA_PIG (P18137) Alpha-lactalbumin precursor (Lactose synthase B protein)	?
Ss1.1-reli1114b_h24.5	eli	72.7190	479	fli	31.4465	155	1.34889e-22	M0	A1AT_PIG (P50447) Alpha-1-antitrypsin precursor (Alpha-1 protease inhibitor) (Alpha-1- antiproteinase)	+
Ss1.1-rssp31_a10.5	fat	17.1016	116	ski	1.9076	13	1.35371e-22			?
Ss1.1-rfat0138_i21.5.5	fat	20.1976	137	ski	3.2282	22	2.72230e-22	M5	Q9H354_HUMAN (Q9H354) PRO1933	?
Ss1.1-rnlu1830b_g11.5	nlu	24.9185	130	elu	1.1561	3	1.50643e-19	M0	PSPC_PIG (P15785) Pulmonary surfactant-associated protein C (SP-C) (Pulmonary surfactant-associated proteolipid SPL(Val))	+
Ss1.1-rton1228b_k22.5	ton	13.5135	77	nep	0.5518	3	1.92833e-19	M2	K2C6A_HUMAN (P02538) Keratin, type II cytoskeletal 6A (Cytokeratin 6A) (CK 6A) (K6a keratin)	+

7 Accession numbers

Table S4: The merged ranges of the accession numbers for the Sino-Danish ESTs sequences available under the center name SDJVP (Sino-Danish Joint Venture Project) at NCBIs trace archive, in the period of July 16 to July 31, 2006.

Range	TI start	TI end
1	1372588356	1372588534
2	1373542512	1373550691
3	1373553740	1373554377
4	1374455571	1374455833
5	1374460033	1374477206
6	1374493724	1374496358
7	1374516358	1374530585
8	1374622420	1374630037
9	1374647814	1374672203
10	1374759941	1374785708
11	1374788781	1374792215
12	1374813074	1374830446
13	1374872458	1374877824
14	1374882145	1374890244
15	1374912158	1374922748
16	1374929853	1374934617
17	1375363597	1375379964
18	1375407255	1375418471
19	1376790949	1376807986
20	1376876515	1376892947
21	1376933435	1376950392
22	1376998303	1377016954
23	1377102633	1377117523
24	1377189604	1377206265
25	1377253980	1377272493
26	1377299975	1377317340
27	1377325201	1377338507
28	1377343692	1377358181
29	1377534935	1377539735
30	1377555096	1377573748
31	1377634710	1377642289
32	1377662532	1377672058
33	1377705896	1377724109
34	1377739944	1377787073
35	1377853725	1377869948
36	1377888668	1377906145
37	1377950861	1377968093
38	1377976734	1377994190
39	1379681547	1379694891
40	1379734210	1379750367
41	1379767659	1379783424
42	1379785709	1379801707
43	1379917493	1379961465
44	1380064442	1380080379
45	1380101383	1380117576
46	1380337508	1380377319
47	1380628018	1380639932
48	1380694786	1380697658
49	1380717658	1380731757
50	1380781374	1380823792
51	1380913793	1380922205
52	1380932206	1380949414
53	1380999415	1381017794
54	1381092978	1381108837
55	1381241046	1381256415
56	1381334730	1381347703
57	1381355288	1381362737
58	1382340999	1382343215
59	1382446420	1382465179
60	1382472780	1382491270
61	1382497855	1382513728
62	1382526627	1382543876
63	1382576521	1382580682
64	1382665335	1382677174
65	1382786748	1382799972
66	1382879973	1382881324
67	1382905125	1382906756
