# Supplementary figures and images for: Porcine transcriptome analysis based on 97 non-normalized cDNA libraries and assembly of 1,021,891 expressed sequence tags
Source: Genome Biol. 2007 Apr 2;8(4):R45. doi: 10.1186/gb-2007-8-4-r45 (PMC1895994; doi:10.1186/gb-2007-8-4-r45)

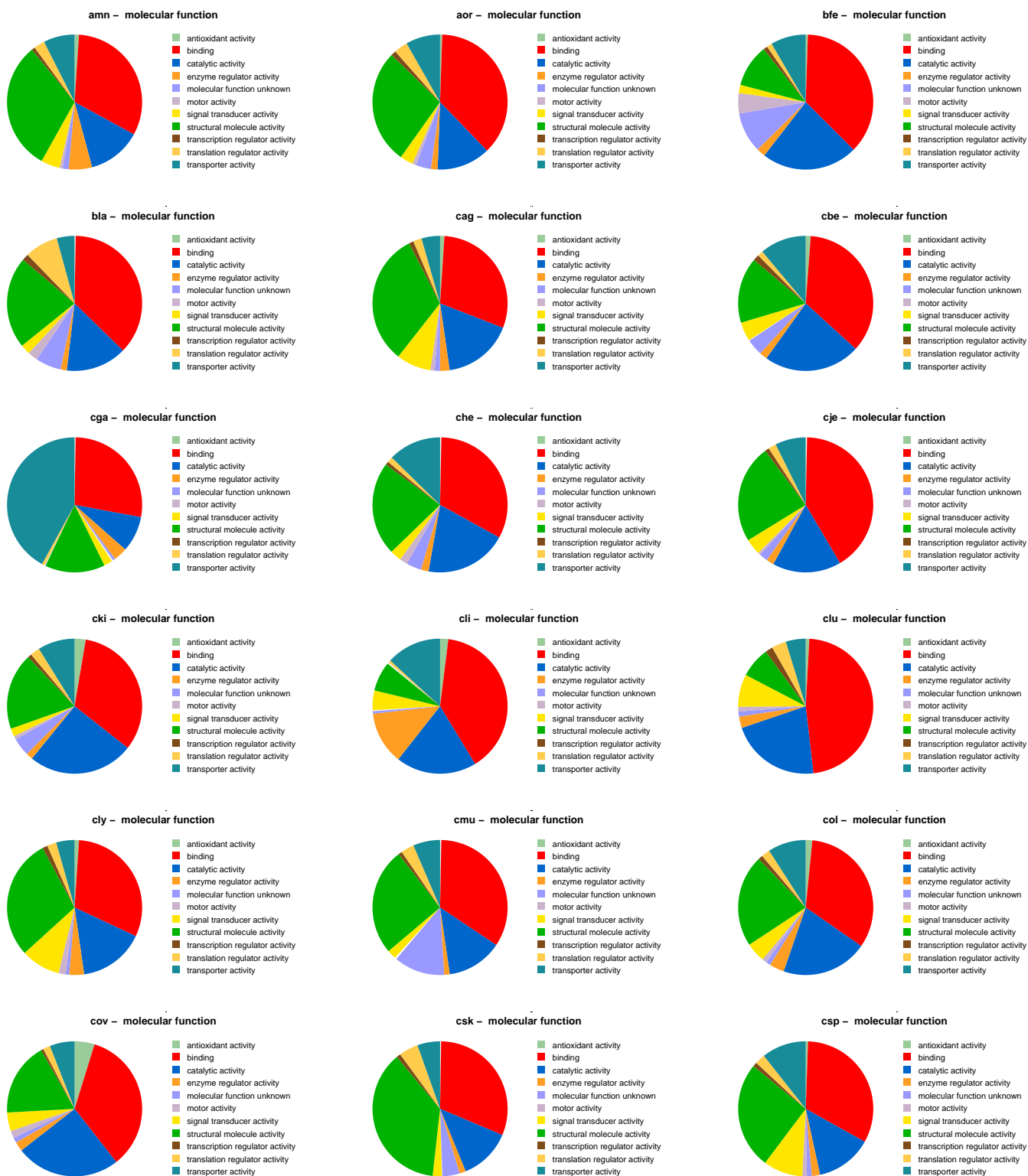



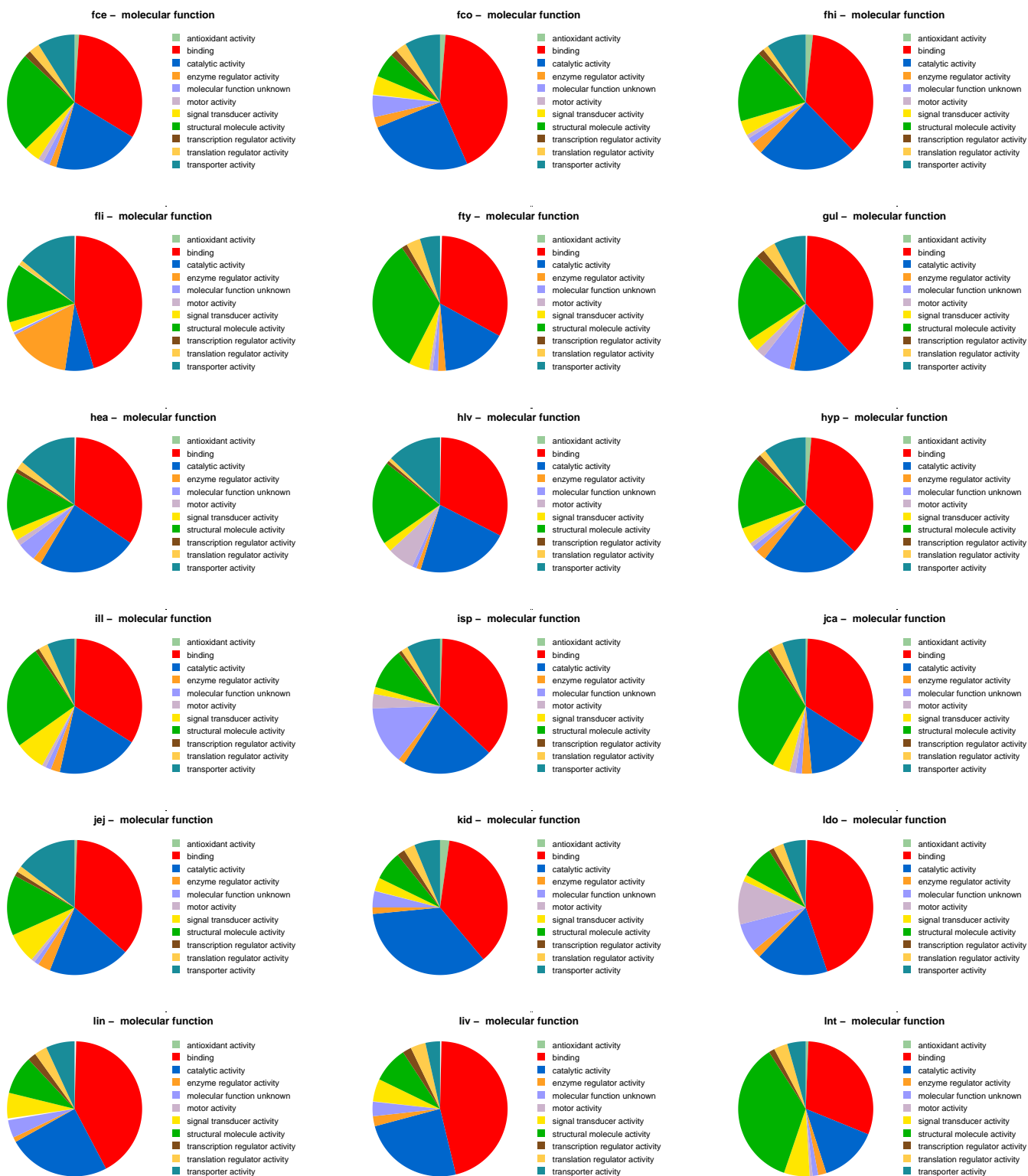

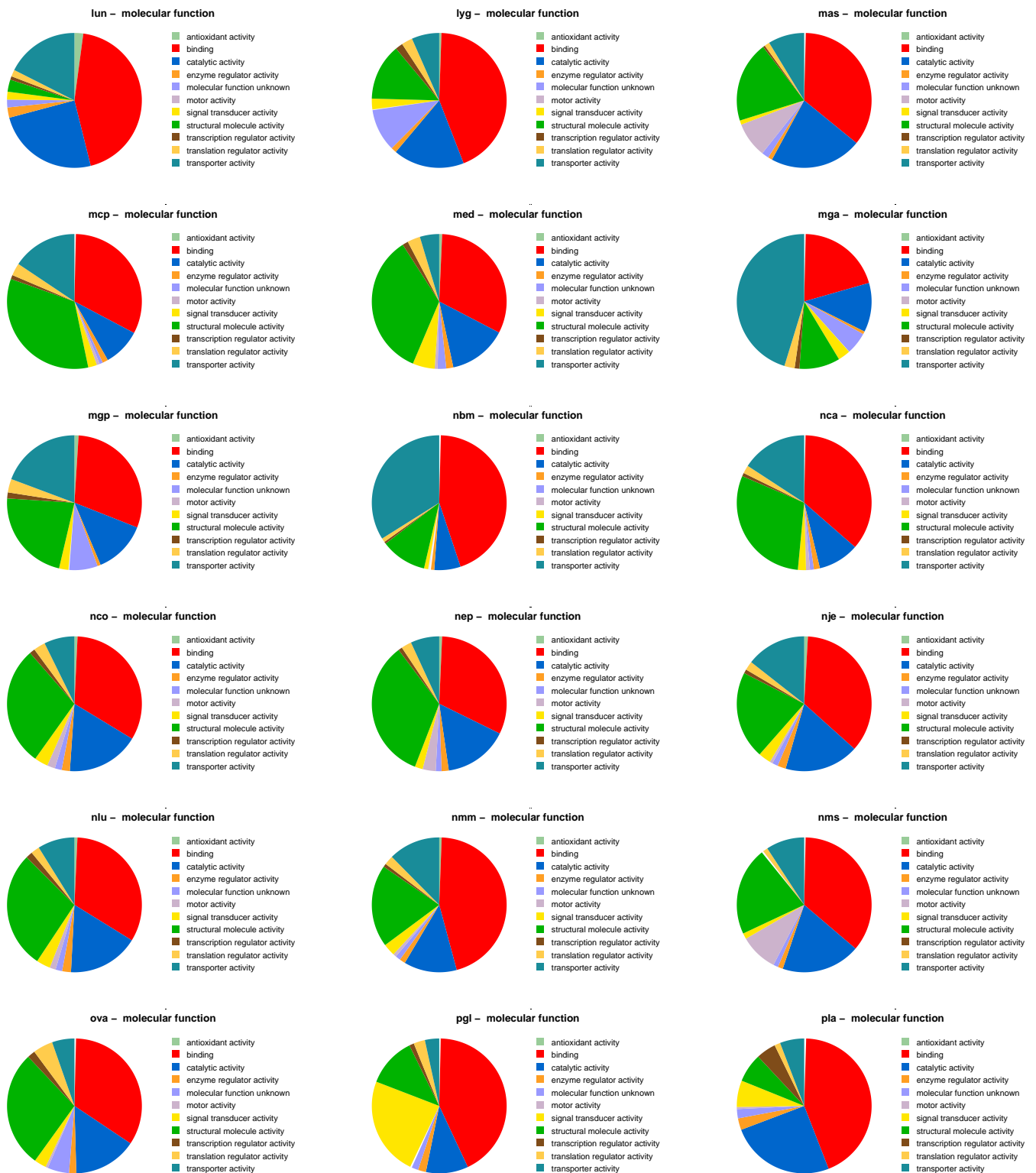

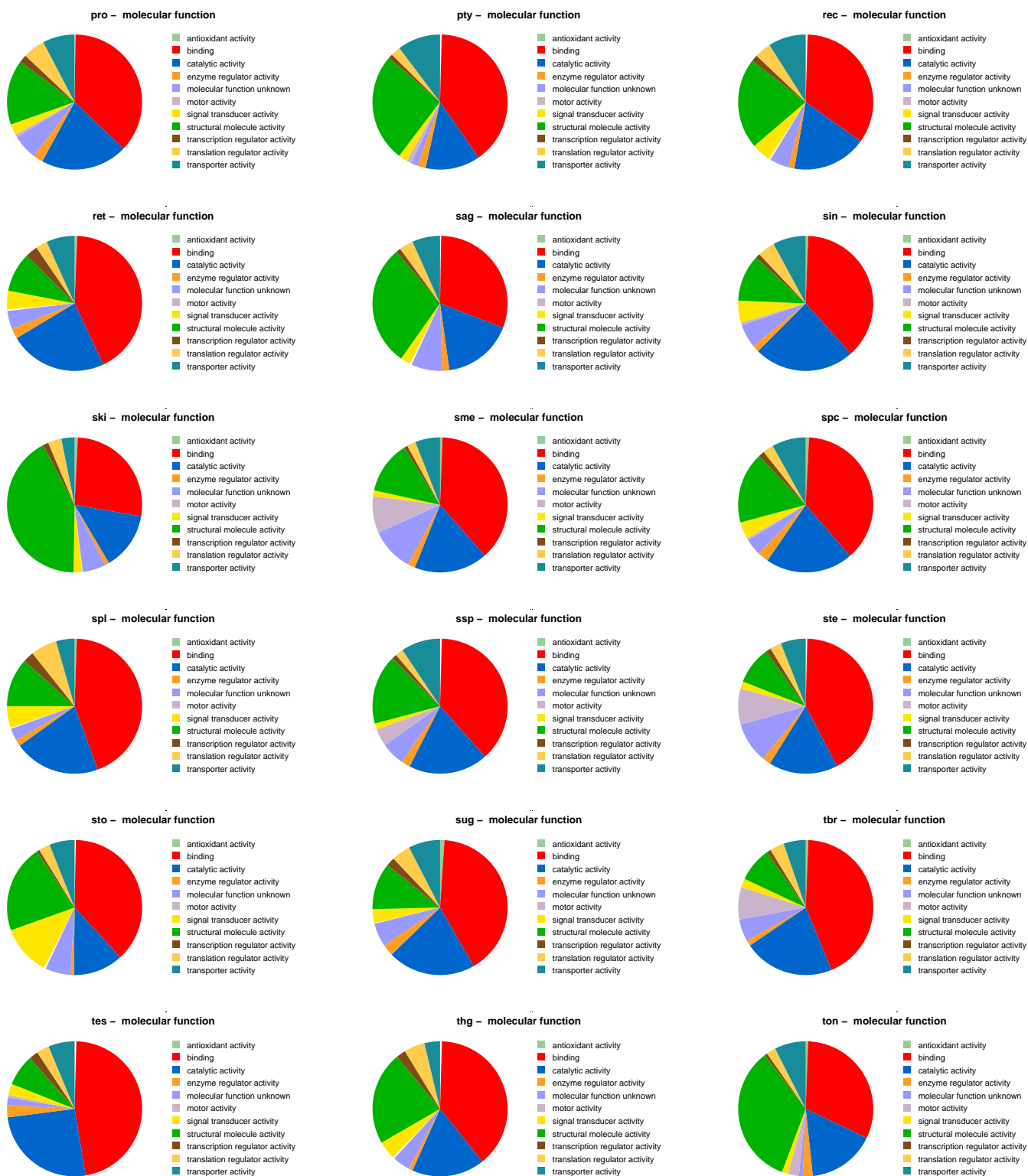

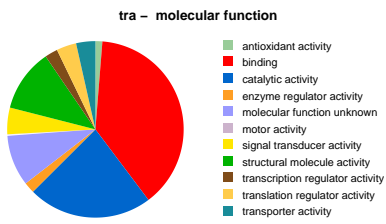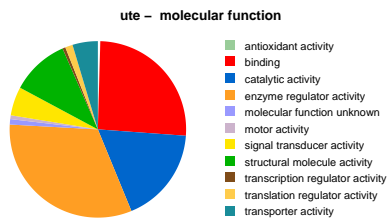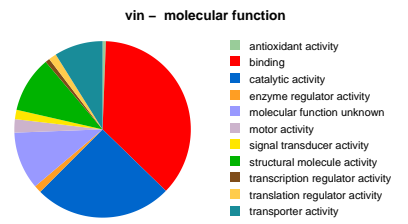

Supplement: Additional data file 2 — contains pie charts of how the expression for each cDNA library is distributed in the main level of GO category 'molecular function'. [file gb-2007-8-4-r45-S2.pdf]

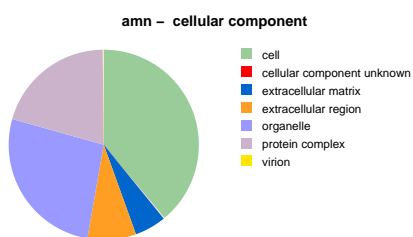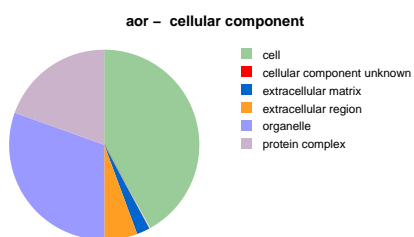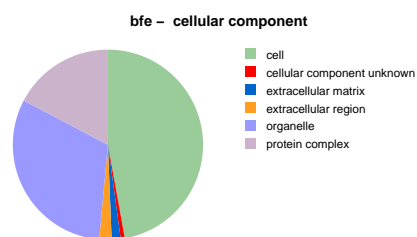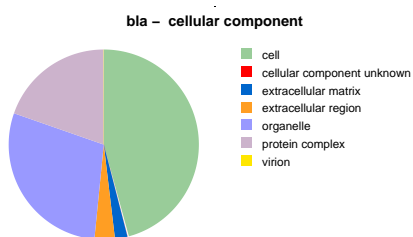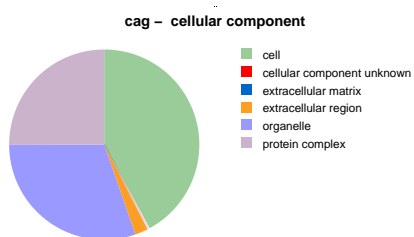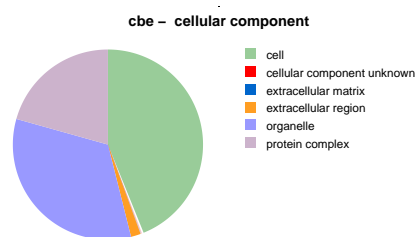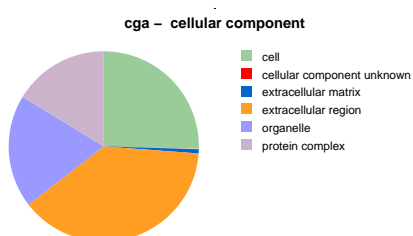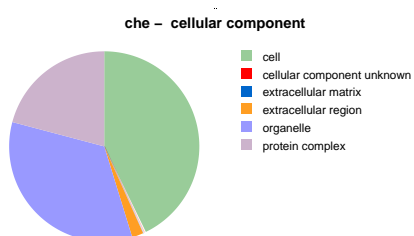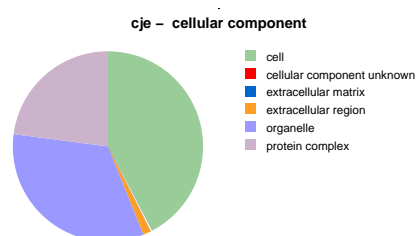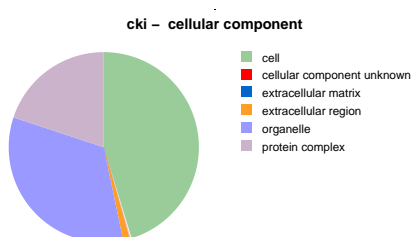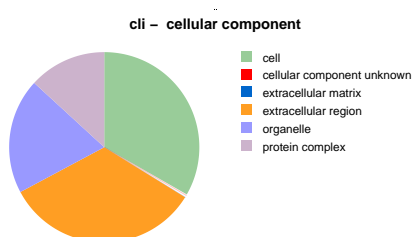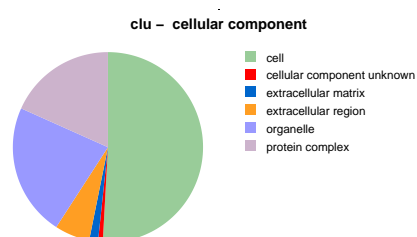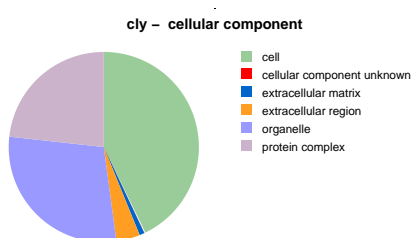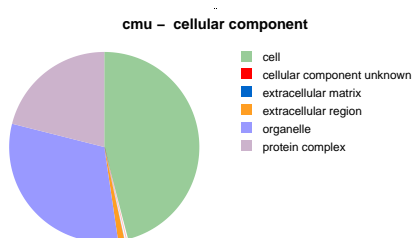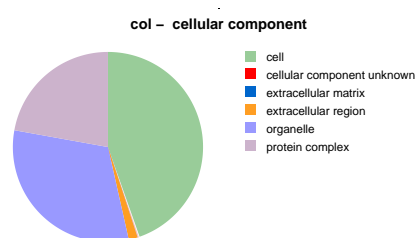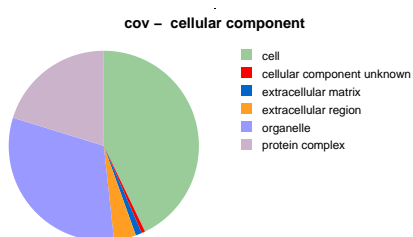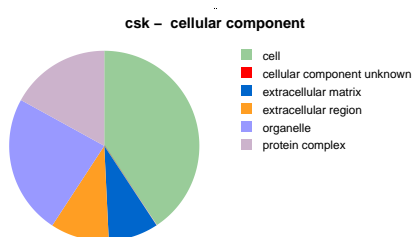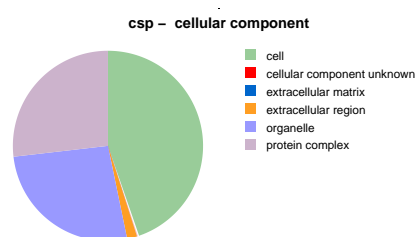

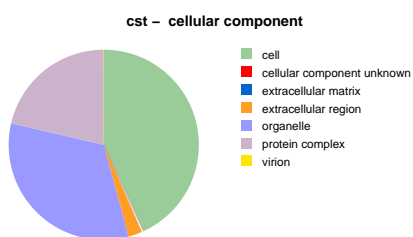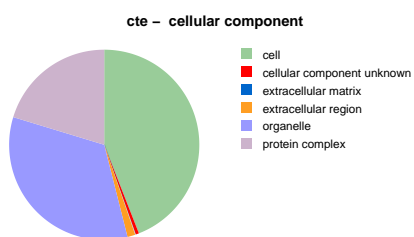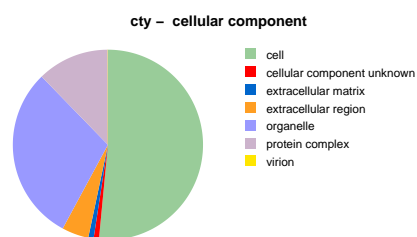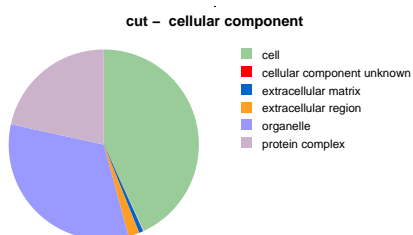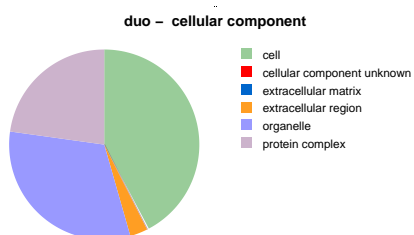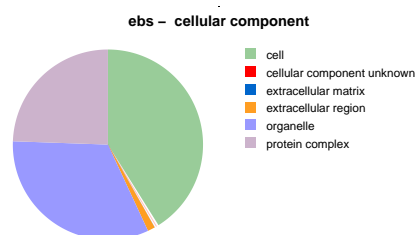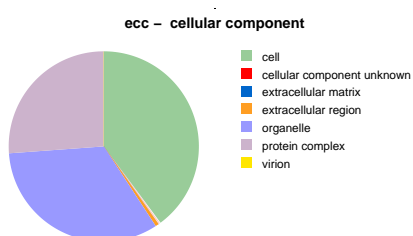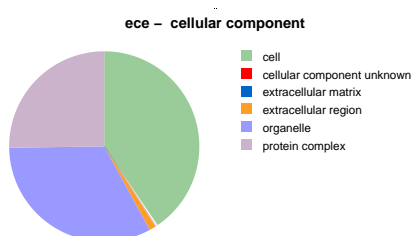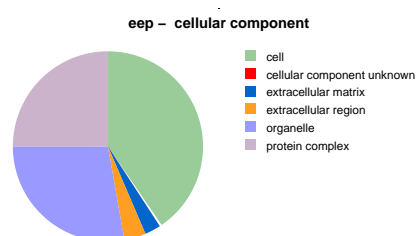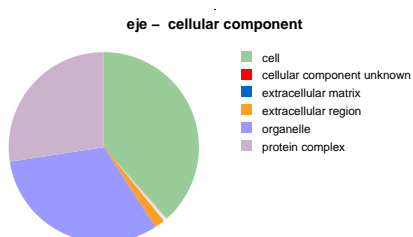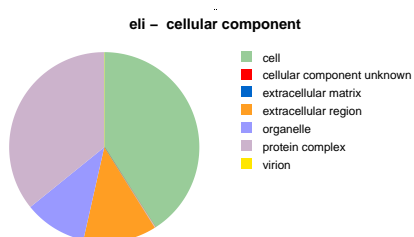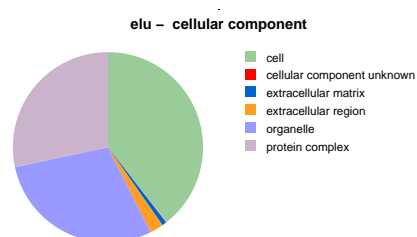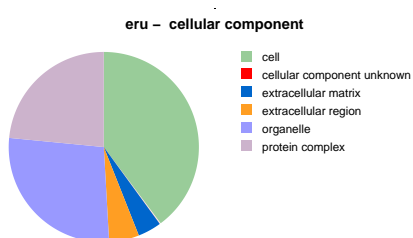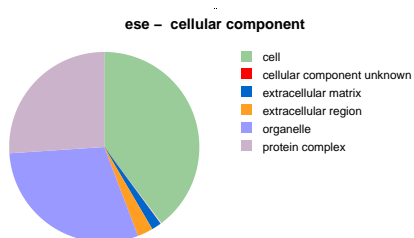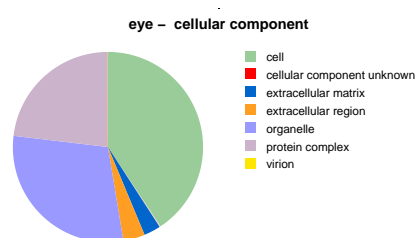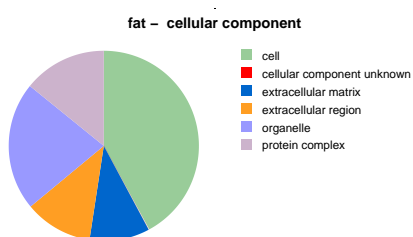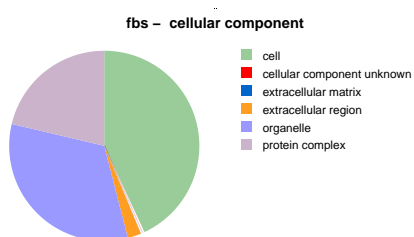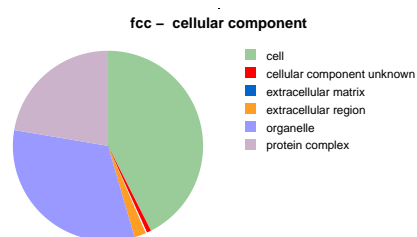

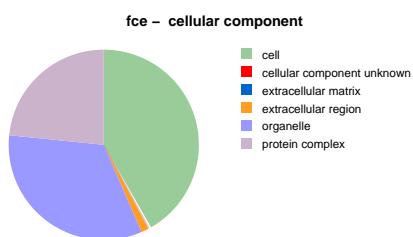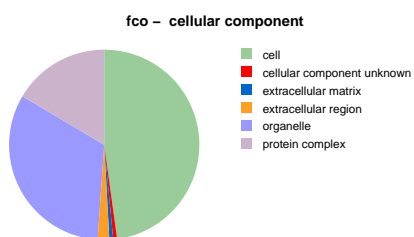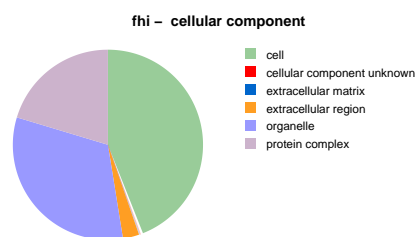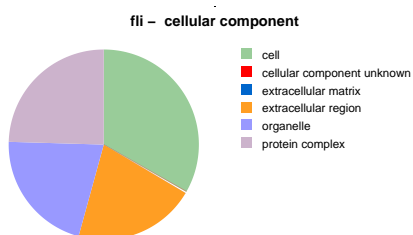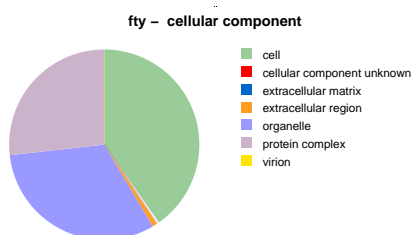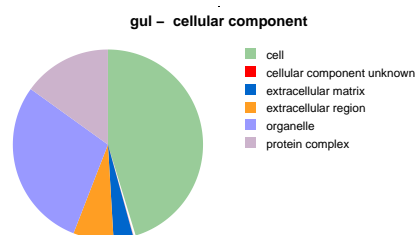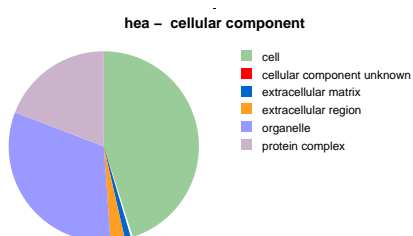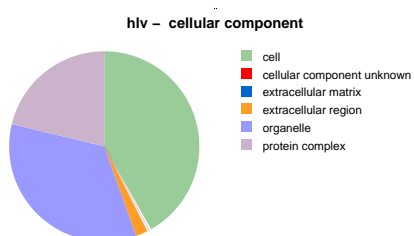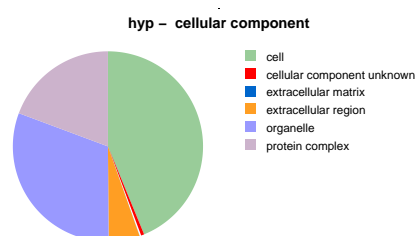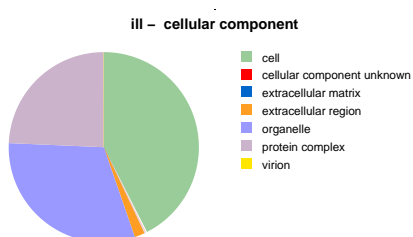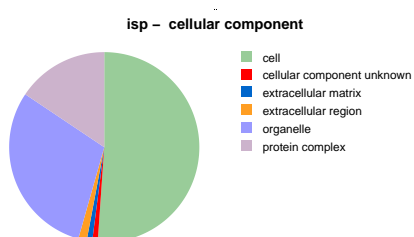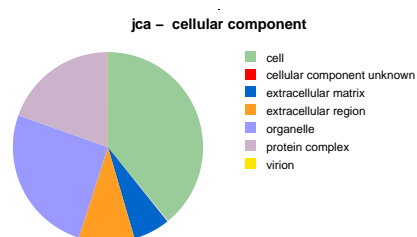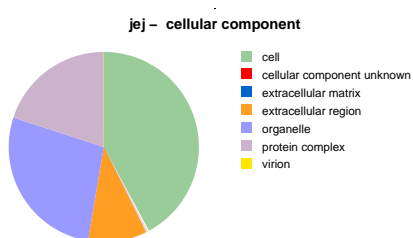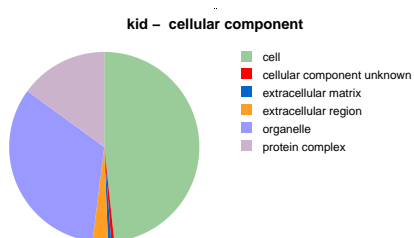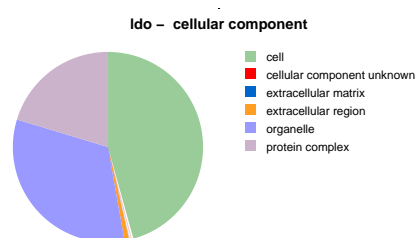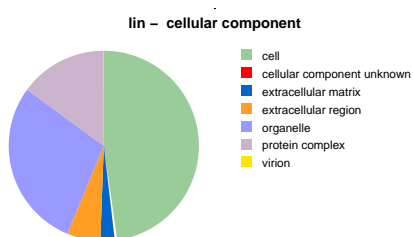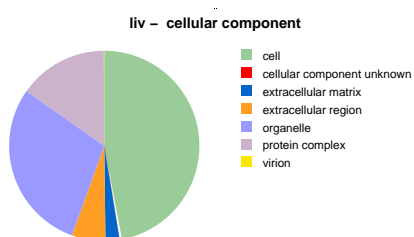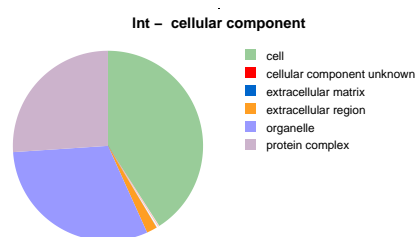

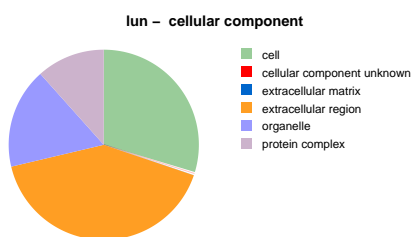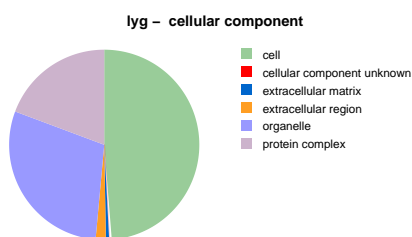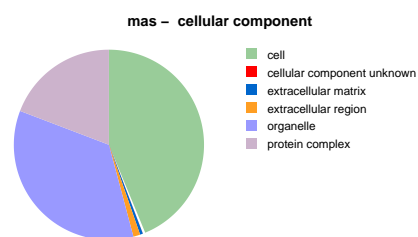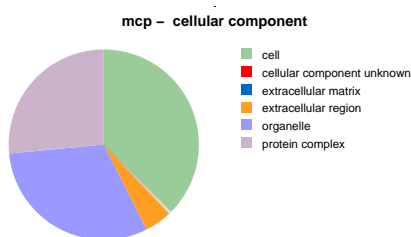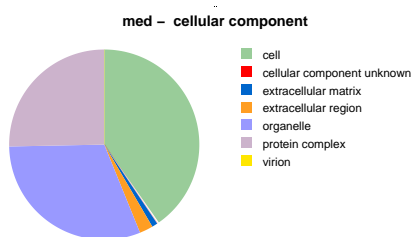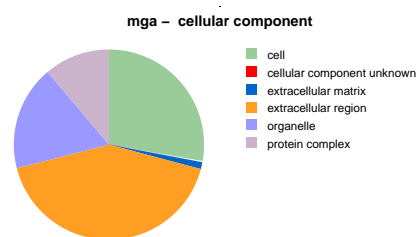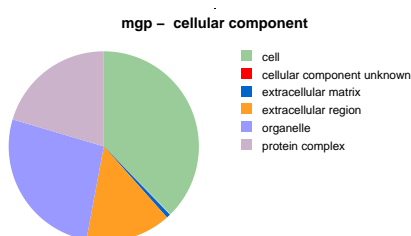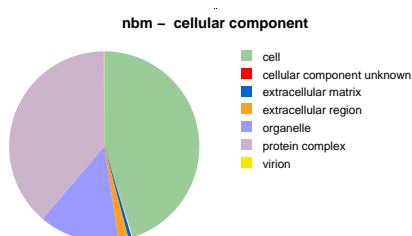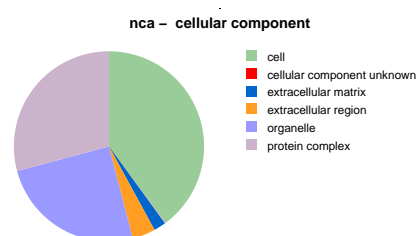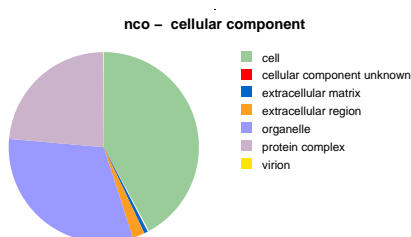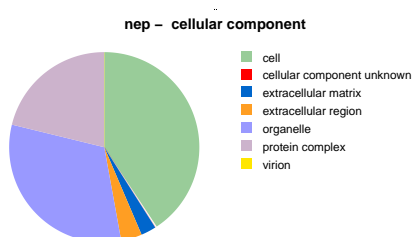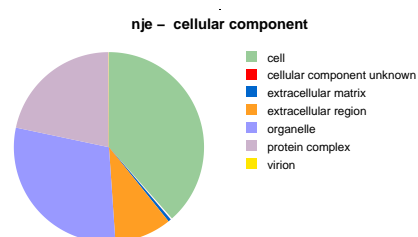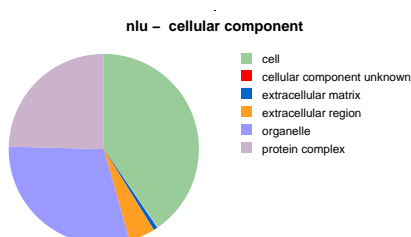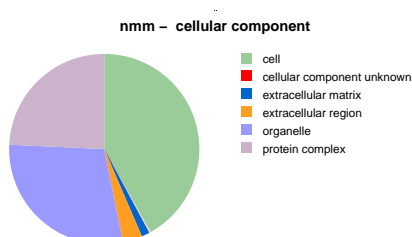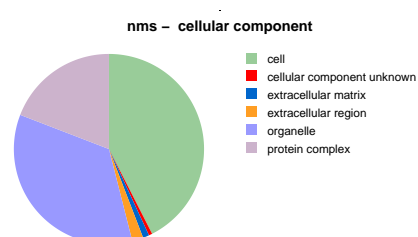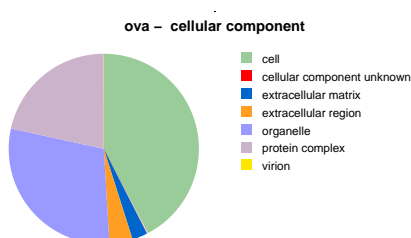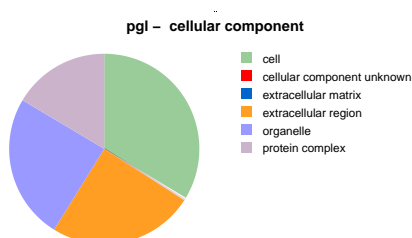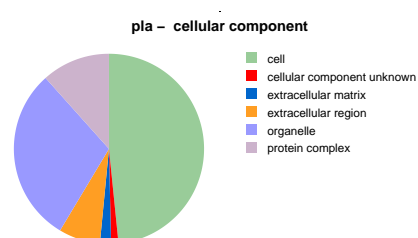

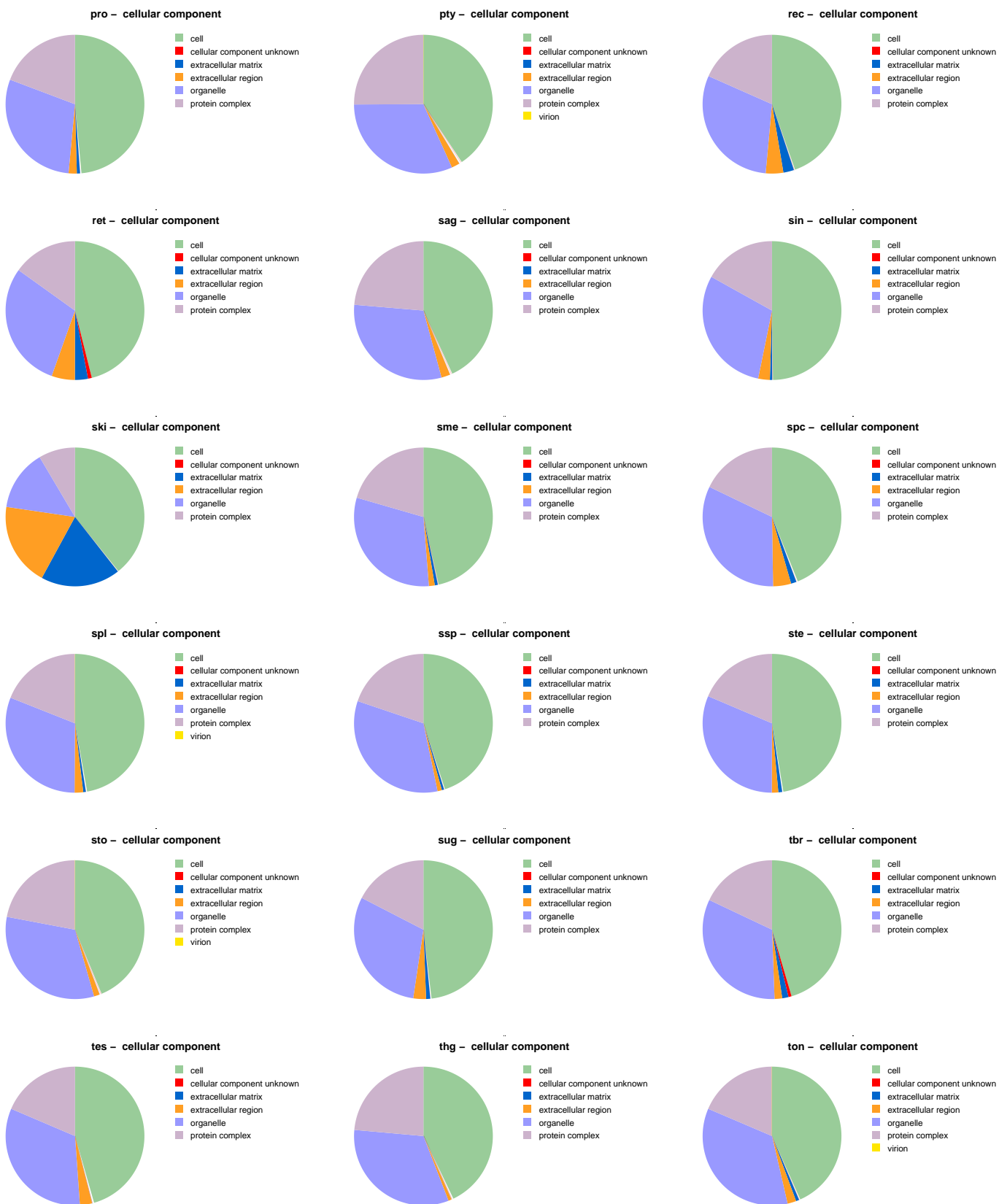

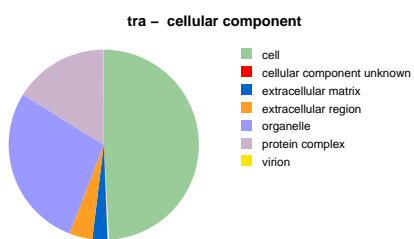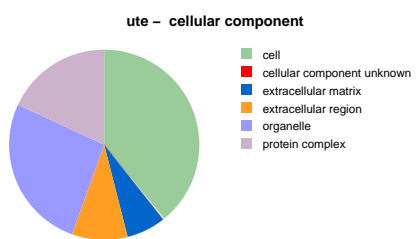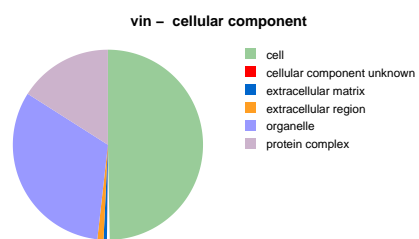

Supplement: Additional data file 3 — contains pie charts of how the expression for each cDNA library is distributed in the GO category of 'cellular component'. [file gb-2007-8-4-r45-S3.pdf]

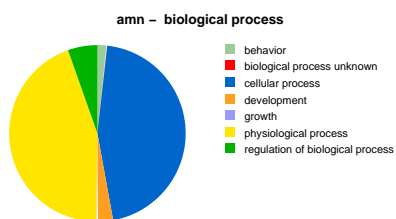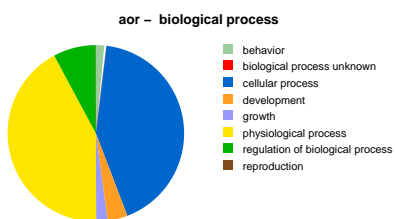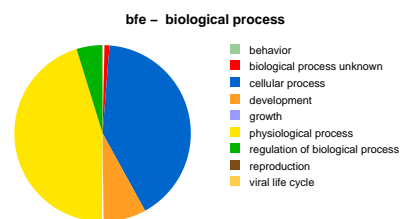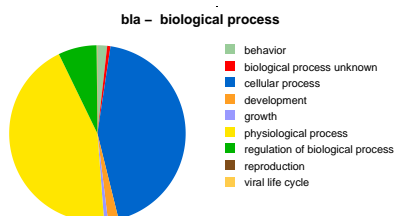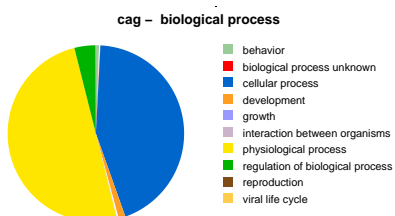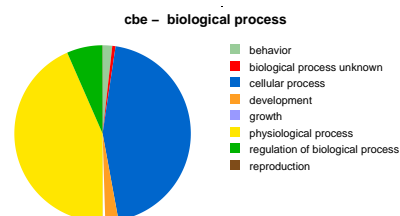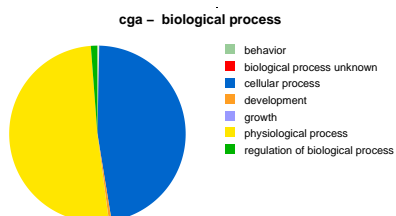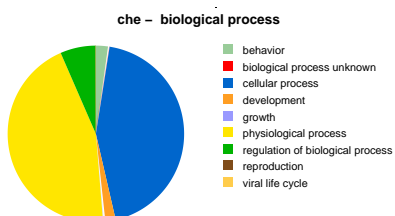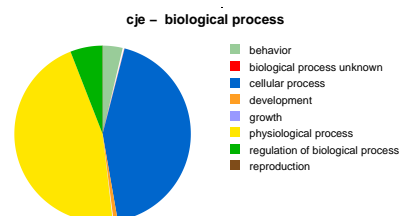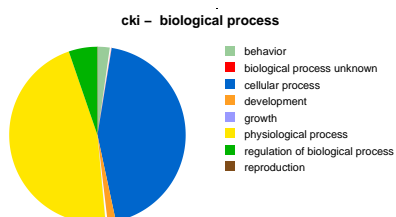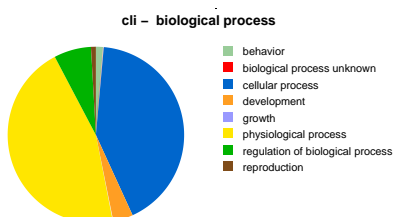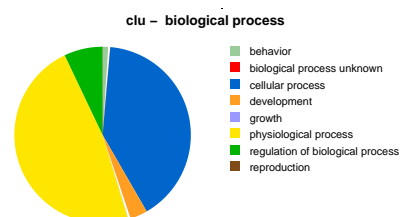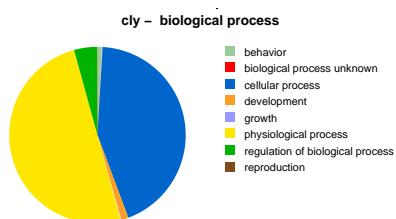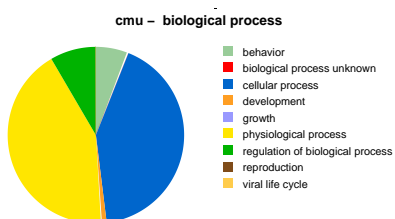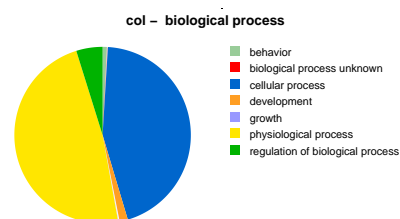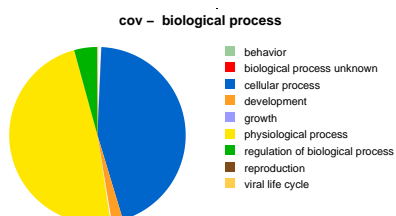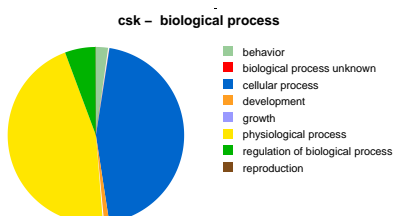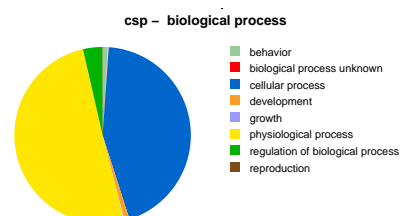

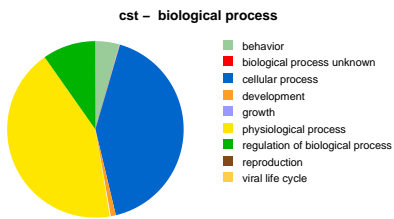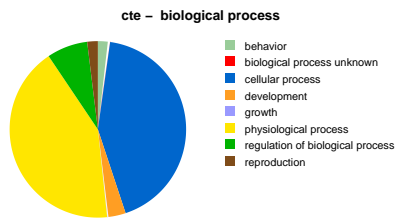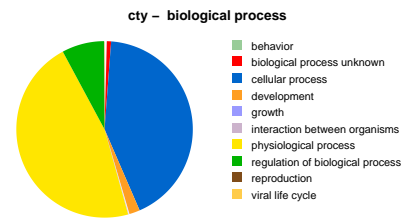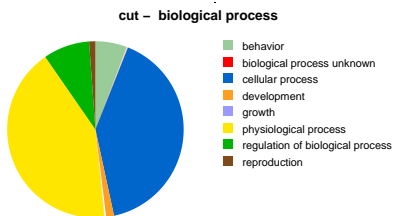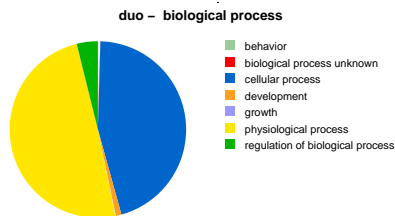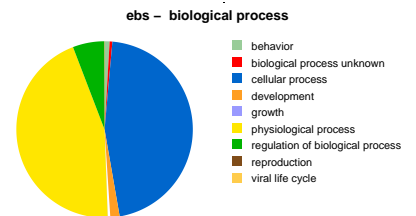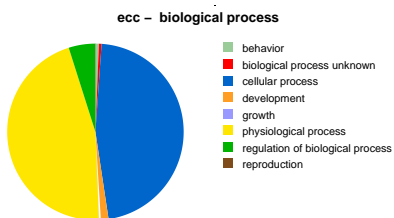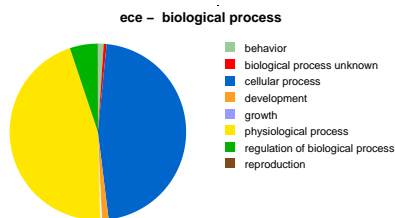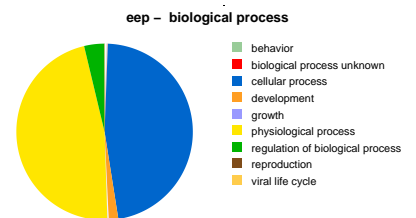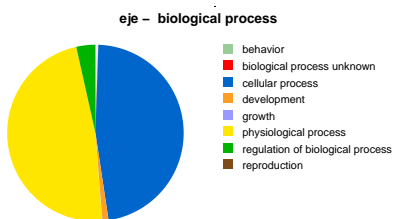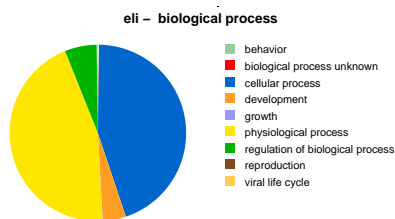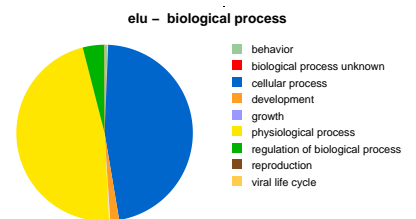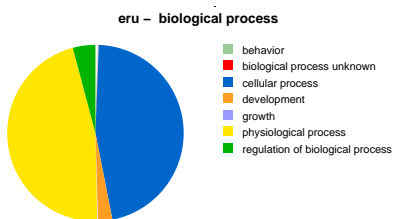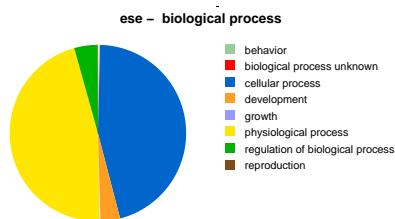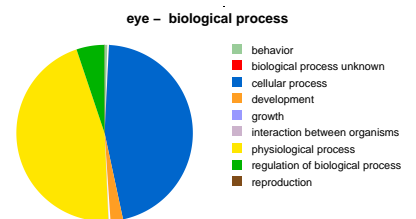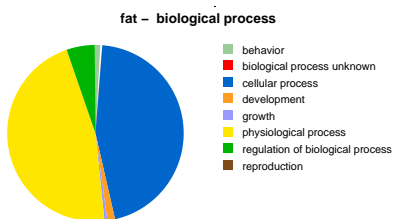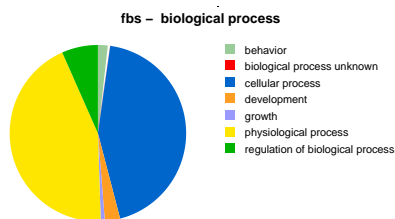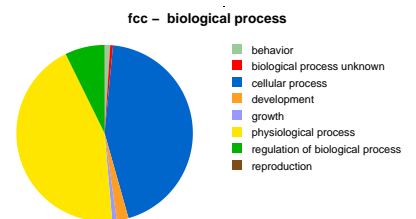

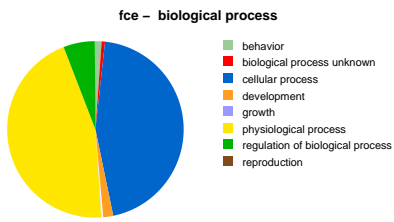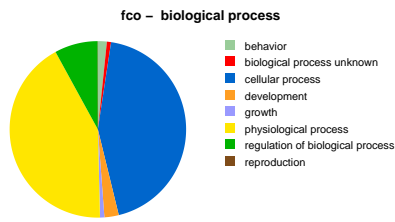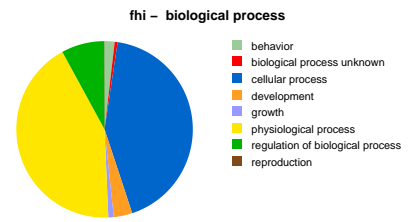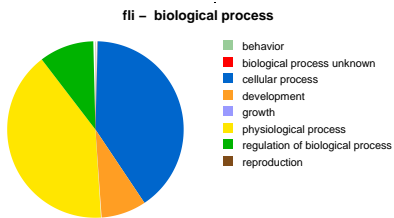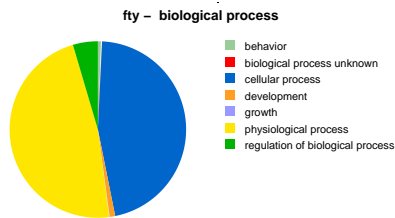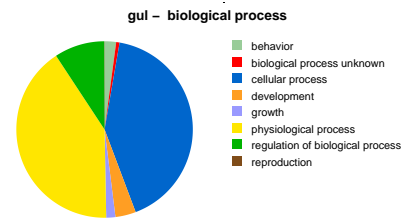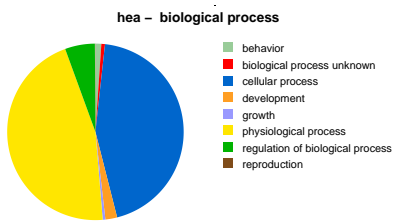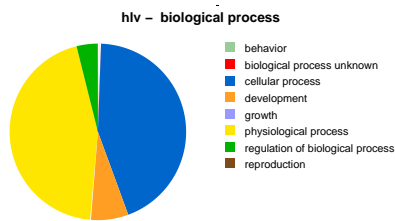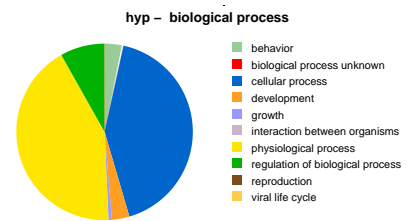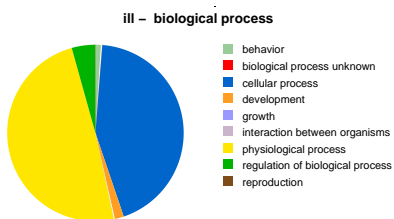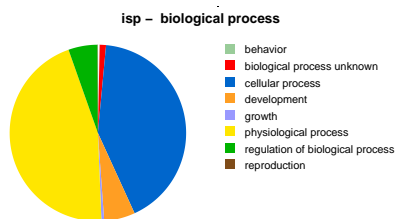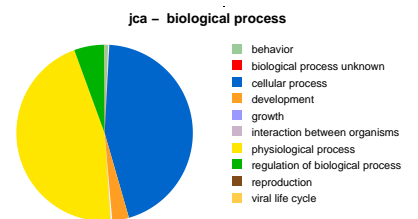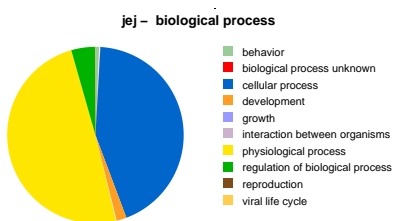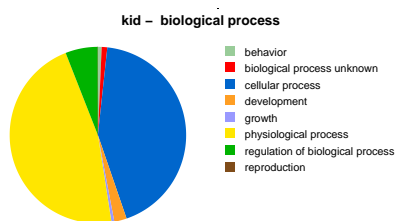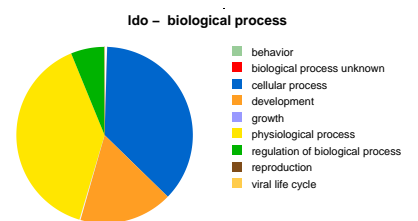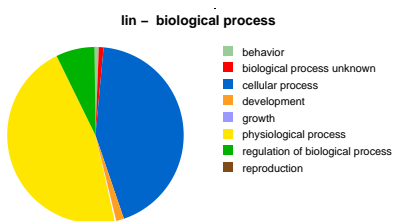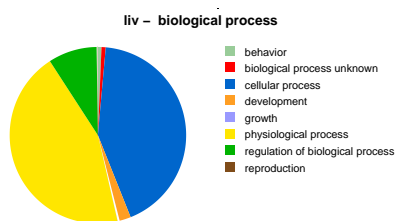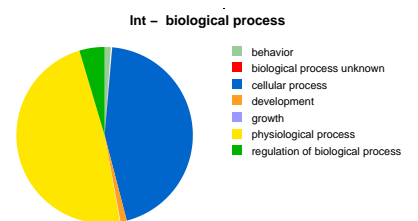

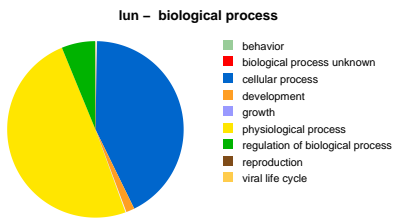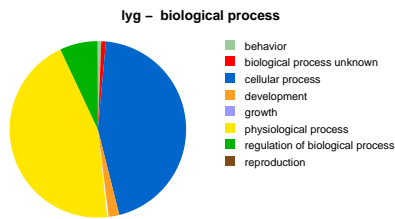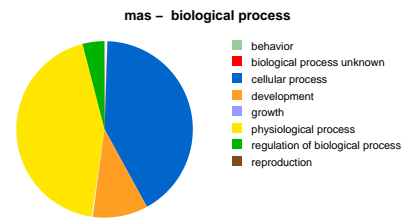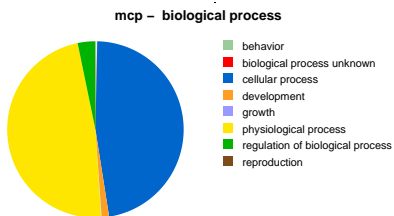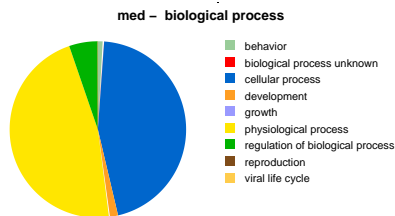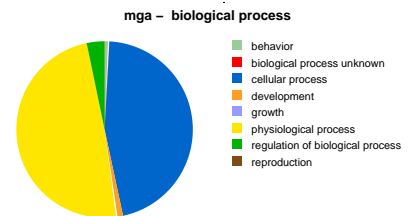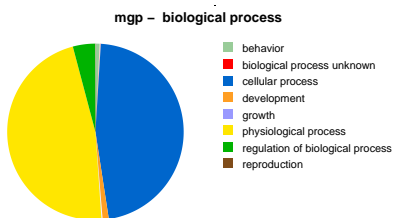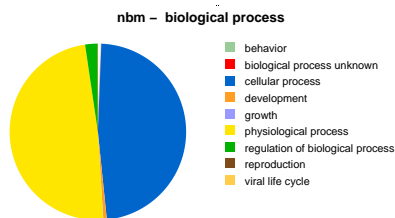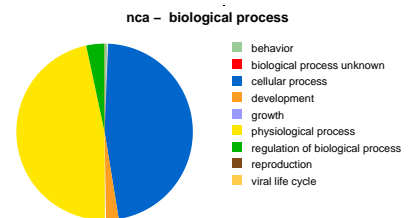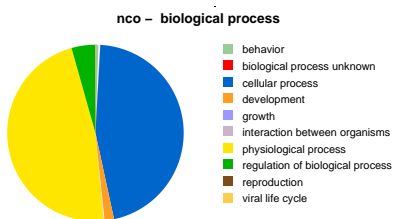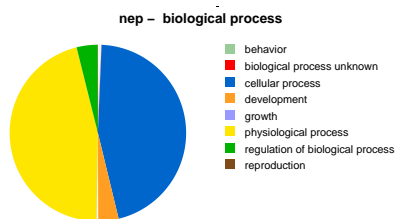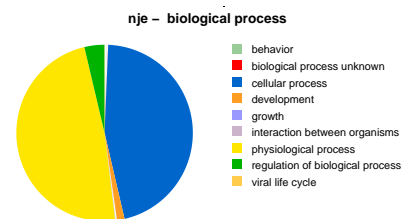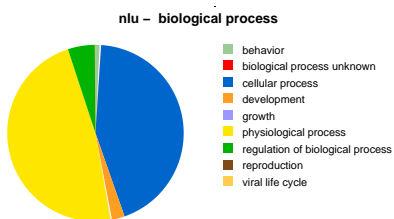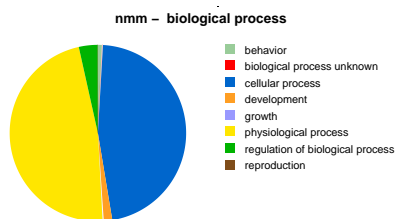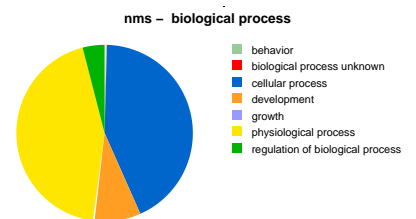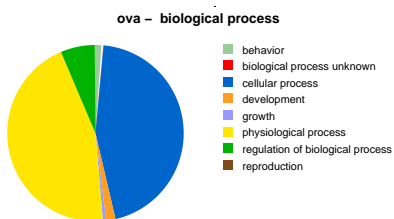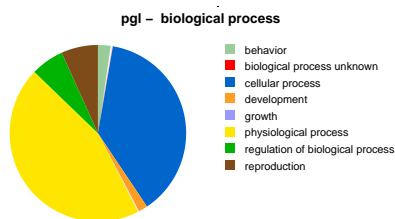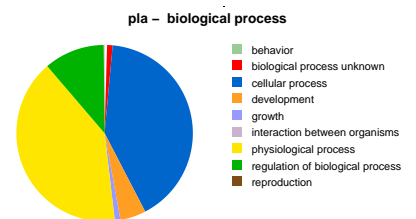

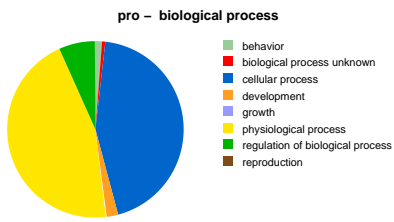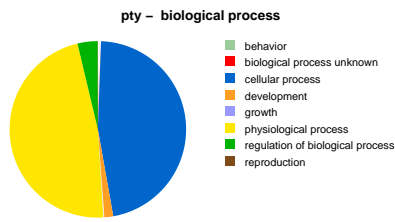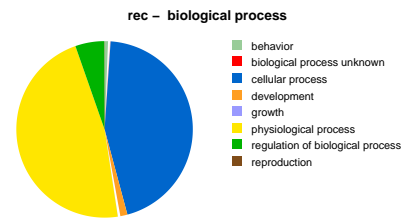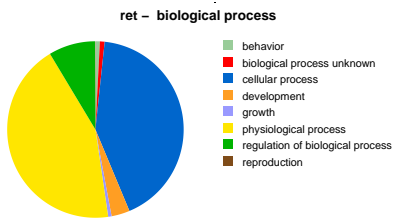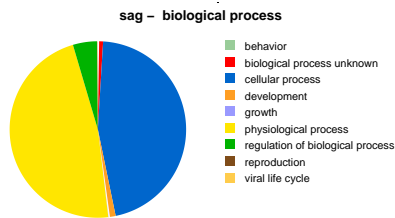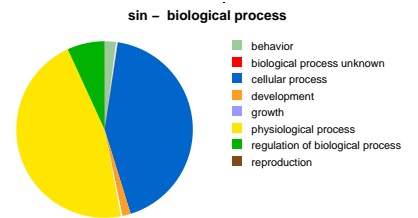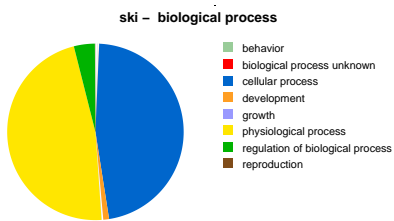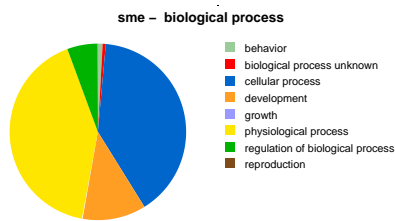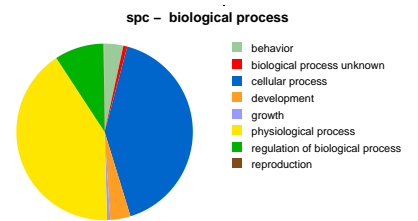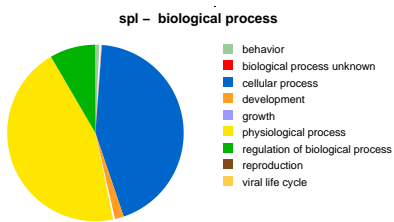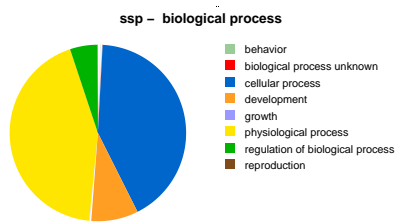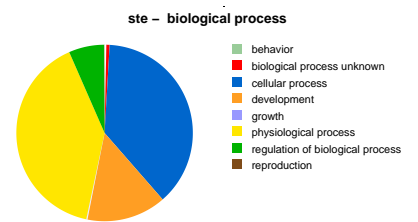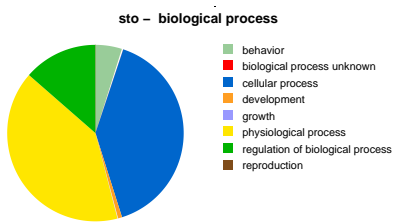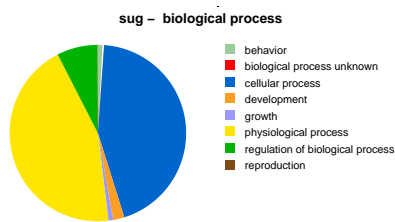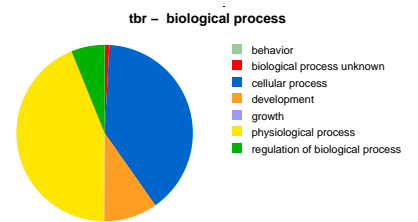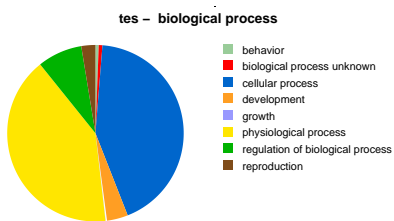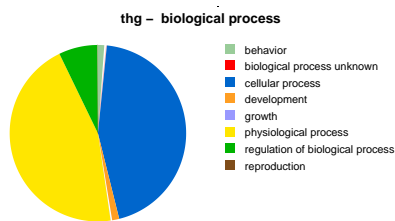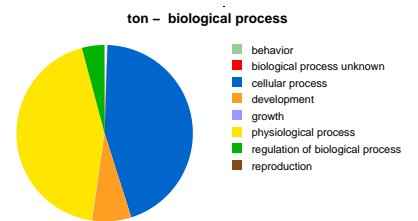

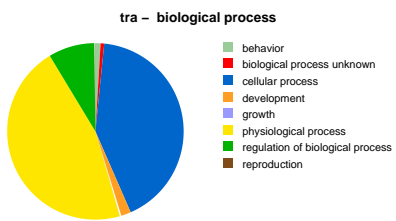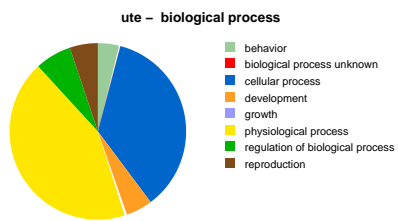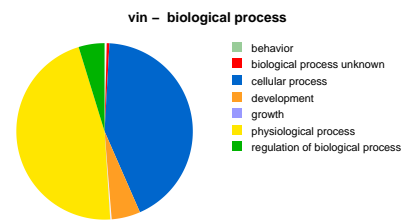

Supplement: Additional data file 4 — contains pie charts of how the expression for each cDNA library is distributed in the GO category of 'biological process'. [file gb-2007-8-4-r45-S4.pdf]
